# Revealing lost secrets about Yingpan Man and the Silk Road

**DOI:** 10.1038/s41598-021-04383-5

**Published:** 2022-01-13

**Authors:** Tingting Wang, Benjamin T. Fuller, Hongen Jiang, Wenying Li, Dong Wei, Yaowu Hu

**Affiliations:** 1 Department of Anthropology, School of Sociology and Anthropology, Sun Yat-Sen University, Guangzhou, 510275 People’s Republic of China; 2 The Needham Research Institute, Cambridge, CB3 9AF UK; 3 Department of Archaeology and Heritage Studies, School of Culture and Society, Aarhus University, 8270 Højbjerg, Denmark; 4 Department of Anthropology and Archaeology, University of Chinese Academy of Sciences, Beijing, 100049 People’s Republic of China; 5 Institute of Archaeology and Cultural Relics of Xinjiang Uyghur Autonomous Region, Urumqi, 830000 People’s Republic of China; 6 College of Archaeology, Jilin University, Changchun, 130012 People’s Republic of China; 7 Institute of Archaeological Science, Fudan University, Shanghai, 200433 People’s Republic of China; 8 Department of Cultural Heritage and Museology, Fudan University, Shanghai, 200433 People’s Republic of China

**Keywords:** Anthropology, Archaeology, Stable isotope analysis

## Abstract

Yingpan Man, is one of the most exquisitely preserved mummies found in the Xinjiang Uyghur Autonomous Region of China. Here links between Yingpan Man and the Silk Road are explored through a detailed isotopic and bioarchaeological investigation of his life history. Analytical techniques of carbon, nitrogen, and sulfur stable isotope ratio analysis on hair, teeth, muscle and bones as well as associated animal and plant remains, radiocarbon dating and starch grain analysis of dental calculus are presented to visualize never before seen aspects of Yingpan Man’s life, including: environment, breastfeeding and weaning practices, adolescent and adult diet, disease and nutritional status as well as season of death. Furthermore, in combination with a detailed review of his associated grave goods, this research examines the social status and identity of Yingpan Man, and demonstrates the profound impact and cultural fusion that the Silk Road had upon the peoples of Xinjiang and Eurasia.

## Introduction

**Figure 1:**
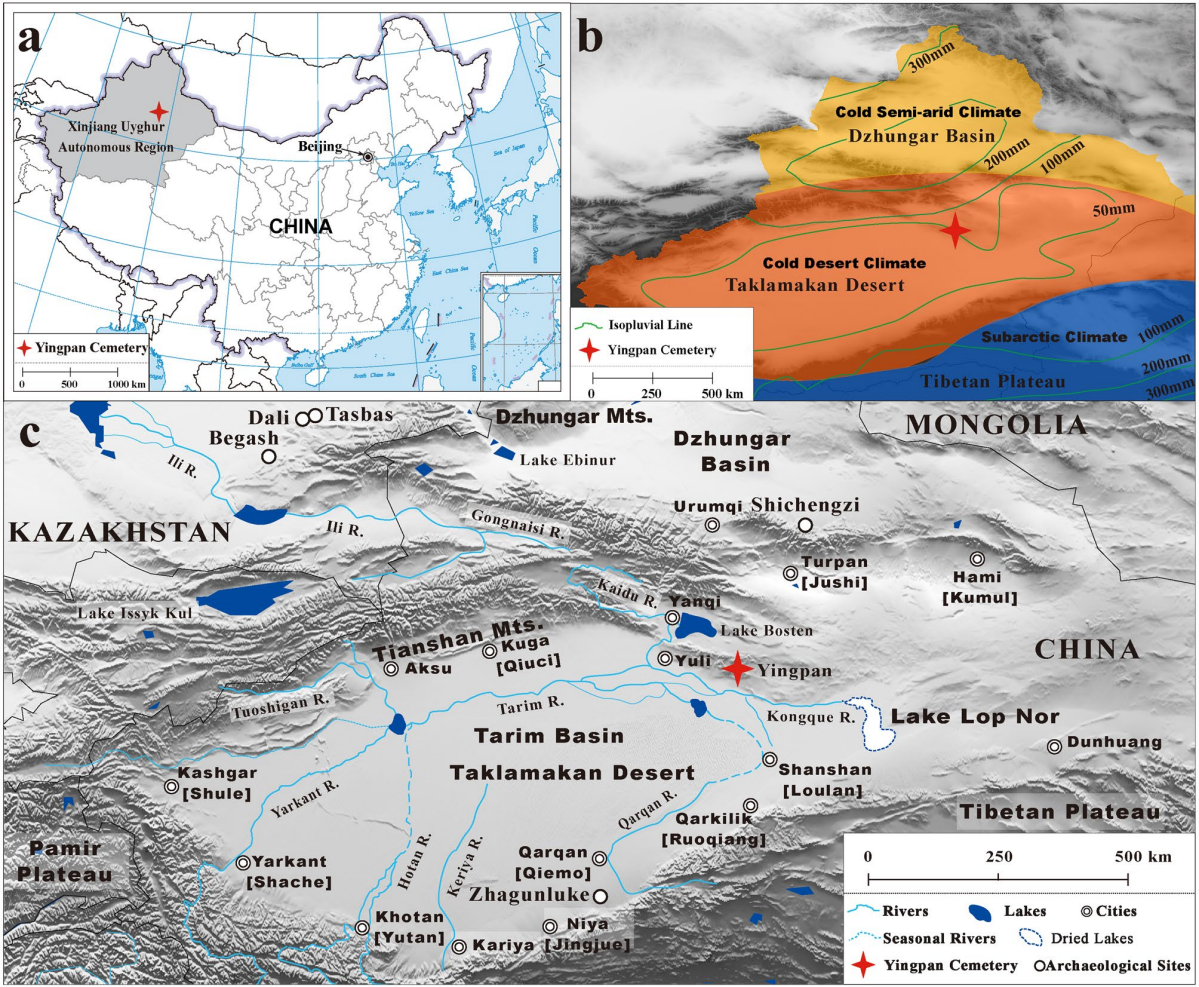
Map of Yingpan cemetery. **(a)** and **(c)** Maps showing the location of Yingpan cemetery (red symbol ) in Xinjiang Uyghur Autonomous Region of China; **(b)** Map showing the distribution of climate zones and isopluvial lines in Xinjiang. (Maps were generated in Standard Map Service (http://bzdt.ch.mnr.gov.cn), GMT 5.2.1 and Global mapper 19. The final layout was created using Adobe Illustrator CC 2019 V.23.1.1.).

The Silk Road was established during the Han Dynasty (202 BC–220 AD)^1,2^. This vast network of trade routes linked China with the west and facilitated an unprecedented increase in bidirectional communication, technology transfer and human movement across Eurasia^3^. Located at a key intersection of this ancient East–West interaction is the modern-day Xinjiang Uyghur Autonomous Region of China (Fig. 1)^4–6^. Abundant archaeological and anthropological research has revealed that the local populations of Xinjiang subsequently experienced dramatic changes in terms of culture and politics and adopted diverse lifeways and economic strategies that were heavily influenced by both aspects of the East and West^4,7,8^. For example, Silk Road caravans transported large amounts of exotic goods and luxuries into the lives of the Xinjiang inhabitants: silk, precious metals, jades, minerals, perfumes, etc.^5,6,8,9^. However, in addition to this economic trade, a remarkably diverse array of ideas, religions, traditions and even diseases were also introduced into ancient Xinjiang^3,10^. In terms of diet, cooking technique and cultigens from different parts of Eurasia were available to the ancient inhabitants of Xinjiang, including wheat (*Triticum aestivum*), barley (*Hordeum vulgare*) and dairy products from the West as well as foxtail (*Setaria italica*) and broomcorn (*Panicum miliaceum*) millet from the East^11–13^.

**Figure 2:**
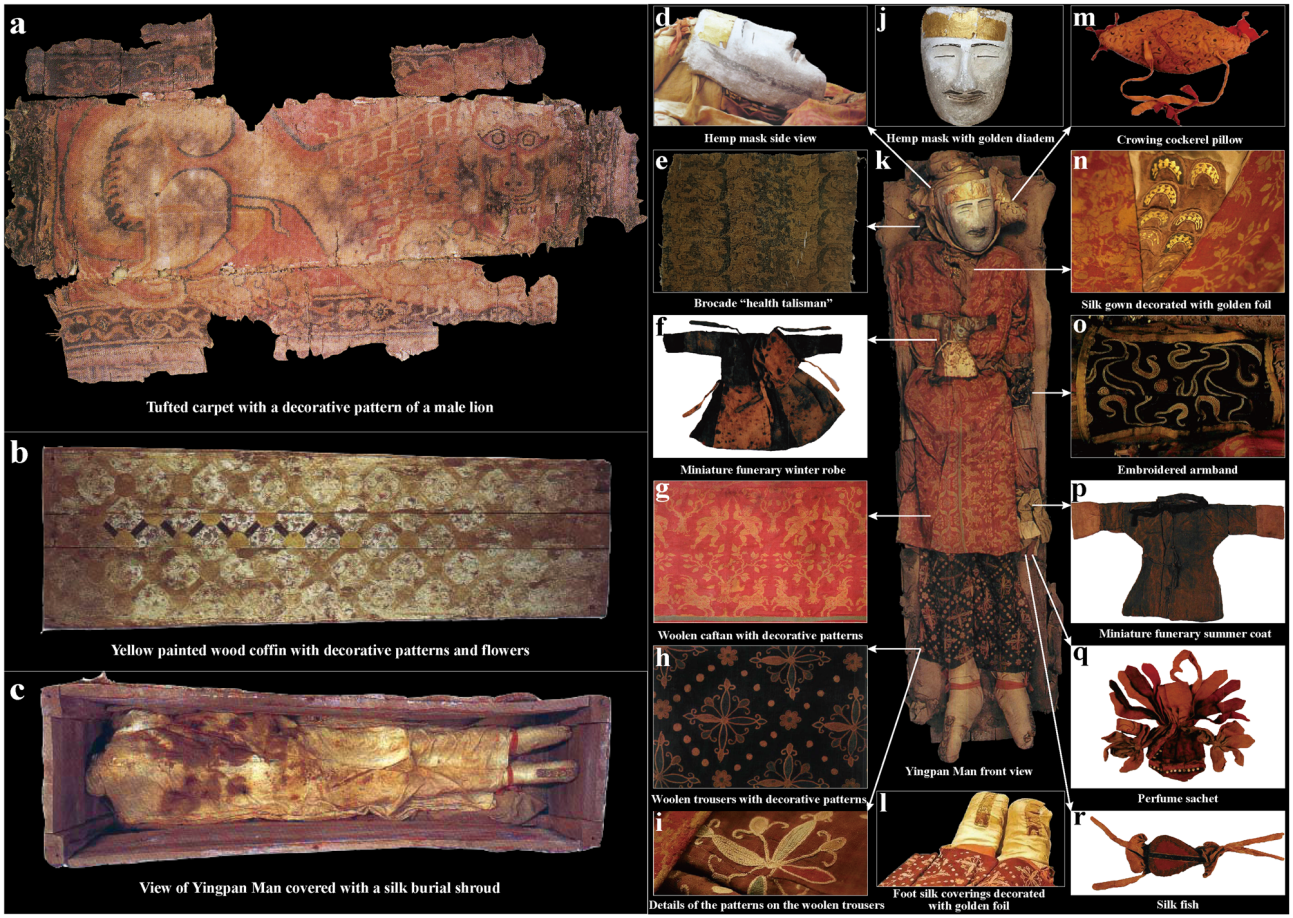
Artifacts of Yingpan Man. **(a)** Image of a tufted carpet with decorative patterns of a male lion. **(b)** Yellow painted wood coffin with decorative patterns of flowers, vines, etc. **(c)** View of Yingpan Man covered with a silk burial shroud. **(d)** Side view of Yingpan Man’s white hemp mask. **(e)** A heavily worn brocade that was decorated with Chinese characters of “Shou” and “You”. **(f)** Miniature funerary winter robe. **(g)** Woolen caftan with decorative patterns of nude putties, animals and pomegranate trees. **(h)** Woolen trousers with decorative patterns of double quatrefoil florals surrounded by lozenges made up of solid circles and flowers. **(i)** Details of the embroidered decorative patterns on the woolen trousers. **(j)** Front view of the white hemp mask with golden diadem. **(k)** Front view of Yingpan Man. **(l)** Foot silk coverings decorated with golden foil. **(m)** Crowing cockerel pillow. **(n)** Silk gown decorated with golden foil on the chest. **(o)** Embroidered armband. **(p)** Miniature funerary summer coat. **(q)** Perfume sachet. **(r)** Silk fish. (The original pictures were previously published^14^ and provided by Wenying Li. The pictures were modified using Adobe Photoshop CC 2015 V.1.2. The final layout was created using Adobe Illustrator CC 2019 V.23.1.1).

Previous archaeological studies concerning the Silk Road were mainly focused on artifacts or the materiality of the Silk Road^8,9^. At present, little research has investigated the past lifeways of individuals that lived along the Silk Road. However, these types of studies permit a better understanding of the nature and influence that this vital network had on populations associated with it^10^. Here, this research seeks to better understand the influence of the Silk Road in Xinjiang through a detailed investigation of the life history of one of the most famous mummies of China: Yingpan Man^14–16^. Specifically, measurements of carbon, nitrogen and sulfur stable isotope ratios on multiple tissues (hair, muscle, bone) and associated animal and plant remains, starch grain analysis of dental calculus and radiocarbon dating are presented here to systematically document and visualize never before seen aspects of Yingpan Man’s life. The extreme arid environment of the Tarim Basin led to the natural desiccation (mummification) of Yingpan Man and to the exceptional preservation of the organic materials and grave goods recovered from his tomb (Figs. 1, 2)^4,7,14^. We review these artifacts in detail and combine this information with the isotopic and bioarchaeological evidence to investigate the social status and identity of one of the most-well preserved and enigmatic mummies recovered from Xinjiang: Yingpan Man. In turn, this work also demonstrates the profound impact and cultural fusion that the Silk Road had upon the peoples of Xinjiang and Eurasia.

## Yingpan Man

In 1995, a tomb containing the remains of an exquisitely preserved male desiccated corpse (mummy) was discovered in the eastern terrace of the Yingpan cemetery located in the Lop Nor region of Xinjiang, China^14^. This individual was called “Yingpan Man”, and based on associated artifacts was believed to date to the Han to Jin Dynasties (202 BC–420 AD) or a period when the Silk Road flourished in this area^14^. He was an exceptionally tall individual measuring ~ 1.9 m in height (the tallest of all the mummies found). His hair was brown and his age at death was approximately 30 years old^14^. While Yingpan Man was buried in a common pit tomb with an upper platform, his grave was exceptional in terms of the quality and diversity of grave goods associated with both the eastern and western portions of the Silk Road^16^. The upper platform of the tomb was covered sequentially with a reed grass-mat (Fig. S1a,f), a layer of grasses consisting of wheat stalks, camel thorn branches (*Alhagi sparsifolia Shap*.), *Sophora alopecuroides* as well as another layer of Populus euphratica sticks (*Populus euphratica Oliv*.) (see Fig. S1a,g). The coffin was draped with an exquisite tufted carpet, elaborately decorated with a colorful pattern of a male lion. This amazing carpet is believed to reflect the high social or political status of Yingpan Man (Fig. 2a)^15^. The coffin itself was made of Populus euphratica wood in a mortise and tenon joint structure, and was ornately painted with geometric patterns of circles and diamonds on all sides as well as decorative patterns of plants, twisted grasses on the cover (Fig. 2b; Fig. S1b,c), flowers, vases, leaves and vines on the sideboards (Fig. S1d,e,h), and pomegranate flowers on the headboard and end-board (Fig. S1i)^14^.

Yingpan Man’s body was covered with a light-yellow silk burial shroud (Fig. 2c; Table S1), his head was resting on a yellow silk pillow in the shape of a crowing cockerel decorated with eight pearls (Fig. 2k,m), and his face was covered with a white hemp death mask containing a golden diadem across his forehead (Fig. 2d,j,k)^14^. He wore a red and yellow woolen caftan that was elaborately embroidered with colorful threads of scenes depicting paired figures of nude puttis, animals (goats and bulls) as well as pomegranate trees (Fig. 2g,k)^14^. In addition, a silk gown decorated with golden foil was worn beneath this caftan (Fig. 2k,n). Yingpan Man’s reddish-purple woolen trousers were chain-stitched embroidered with double quatrefoil floral patterns surrounded by lozenges made of solid circles and flowers (Fig. 2h,i,k)^14^. He also wore a pair of funerary felt socks with silk coverings and golden decorations (Fig. 2k,l)^14^. In addition, a perfume sachet and a silk fish were attached to Yingpan Man’s silk belt (Fig. 2k,q,r), and an embroidered armband was tied around his left arm (Fig. 2k,o), a well-used or frayed brocade decorated with Chinese characters was place on the right side of his head (Fig. 2e,k), a miniature funerary robe was placed on his chest (Fig. 2f,k), while a miniature funerary coat was placed nearby his left wrist (Fig. 2k,p)^14^. Furthermore, Yingpan Man’s nose was filled with T-shaped textiles that were wrapped with silk threads and decorated with golden foil^14^. Beneath his clothes, Yingpan Man’s entire body was found carefully wrapped with soft silk textiles, including his head, his arms, his legs and even each of his toes^15^. Remarkably, a human-shaped wooden board was found on the back of Yingpan Man’s body, and this was fixed tightly on his back with the silk textiles. This lavish but unmarked tomb filled with an array of exquisite artifacts but without any common or everyday living objects, which were popular in the other burials of the Yingpan cemetery, is unprecedented and sets this tomb apart from others found in China from this region and time period^14–17^. As such, there has been much controversy and speculation about the social identity of Yingpan Man: whether he was a wealthy merchant from the West^7^, or a military official from the government of Central China^15^, or possibly a local noble or even a king of the nearby state of Shan^14,16^? These questions and debate make the life history of Yingpan Man a particularly important research topic to be investigated and explored.

## Results

### Radiocarbon date for Yingpan Man

The extracted collagen from the patella of Yingpan Man produced a radiocarbon date of 1730 ± 30 BP (BETA-416250) (Table S2). This corresponds to a 2-sigma calibrated range of 245 to 385 cal AD with a median age probability of 305 cal AD. This age range indicates that Yingpan Man lived during the Jin Dynasty (266 to 420 AD). This is consistent with the funerary artifacts found in the Yingpan cemetery and in his tomb^16^.

### Starch grain analysis

A total of 15 starch grains were recovered from the dental calculus of Yingpan Man’s canine. Among which, 6 were destroyed and were unidentifiable (Fig. S2 a1, a2), while the remaining 9 starch grains were identified to be from two different taxa of plants (Fig. S2). Eight of these starch grains were found to be shaped in a polygon with centric and open hila, radial fissures but no lamellae (Fig. S2 b1, b2, c1, c2). The length of the major axis was between 6.6 and 22.4 μm, and the length of the minor axis was between 5.1 and 16.7 μm. According to published data, starch grains with polygonal shapes are usually characteristic of seeds from the grass family, *Poaceae*^18,19^. Given the fact that broomcorn millet was commonly found at Yingpan cemetery^20^, the polygonal starch grains from Yingpan Man’s canine are likely from his consumption of broomcorn millet.

One starch grain displayed different characteristics by showing a flat and circular form with centric and closed hila on its stationary surface (Fig. S2 d1, d2). The length of the major axis is 21.2 μm, while the length of the minor axis is 20.5 μm. This starch grain has visible lamellae and craters, but no obvious fissures in the front view. However, it was observed to have an oval shape with a longitude fissure from the side view after compression and rotation. When this starch grain was compared with the modern reference collections and published literature, it was found to be consistent with *Triticea*^18,19,21^, or wheat/barley which has been found in other burials from the Yingpan cemetery^20^.

### Plant isotope results

**Figure 3:**
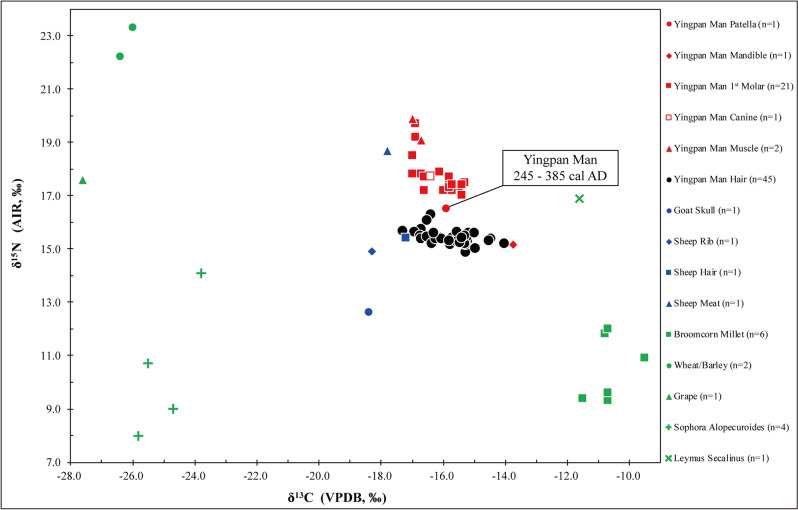
Stable carbon and nitrogen isotope results of multiple body tissues from Yingpan Man and associated animal and plant remains from Yingpan cemetery.

Grains, leaves and stalks from the pillow filler of other burials in the Yingpan cemetery were identified as the wild grass *Sophora alopecuroides* (Fig. S3c)^20^. These were isotopically measured and the δ^13^C results show typical C_3_ signatures and range from − 25.8‰ to − 23.8‰ (mean ± SD = − 24.9 ± 0.9‰), and the δ^15^N values range from 8.0‰ to 14.1‰ (mean ± SD = 10.4 ± 2.7‰) (Fig. 3; Table S3 & S4). Another species of wild plant was identified as the C_4_ wild grass *Leymus secalinus* (Fig. S3d)^20^. A single leaf was isotopically analyzed, and the δ^13^C result was − 11.6‰, and the δ^15^N result was 16.9‰ (Fig. 3; Table S3). It is remarkable that these two species of wild plants display highly elevated δ^15^N values and a large range of environmental isotopic variability.

Eatable plants such as broomcorn millet, wheat/barley, and grape were also analyzed. Pieces of stalk, caryopsis, palea, lemmas and the spikes of broomcorn millet (n = 6, see Fig. S3b) were measured separately and produced δ^13^C values that range from − 11.5‰ to − 9.5‰ (mean ± SD = − 10.6 ± 0.6‰) and δ^15^N values that range from 9.3‰ to 12.0‰ (mean ± SD = 10.5 ± 1.2‰) (Fig. 3). Stalks of wheat/barley (n = 2) showed δ^13^C values of − 26.0‰ and − 26.4‰ as well as δ^15^N values of 23.3‰ and 22.2‰ (Fig. 3). For the wheat/barley, the δ^13^C results were as expected for modern/archaeological plants^22^, but the δ^15^N values were extremely high, though not unprecedented for δ^15^N plant results from Xinjiang^23^. A grape pulp was also isotopically analyzed, yielding a ^13^C-depleted value of − 27.6‰ and a ^15^N-enriched value of 17.6‰ (Fig. 3; Table S3).

### Faunal isotope results

The goat collagen isotopic results were the following: δ^13^C (− 18.4‰), δ^15^N (12.6‰) (Fig. 3; Table S5). The sheep collagen isotopic results yielded the following values: δ^13^C (− 18.3‰), δ^15^N (14.9‰). The hair from the sheep produced similar isotopic results as the collagen: δ^13^C (− 17.2‰), δ^15^N (15.4‰), δ^34^S (9.0‰). The muscle tissue of the sheep had a δ^13^C value of − 17.8‰ and a δ^15^N value of 18.7‰. These faunal isotopic results are similar to plants from the Yingpan cemetery. The sheep and the goat were mainly consuming C_3_ plant proteins with only a minor input of C_4_ plants in their diets.

### Dentine serial sections of Yingpan Man

**Figure 4:**
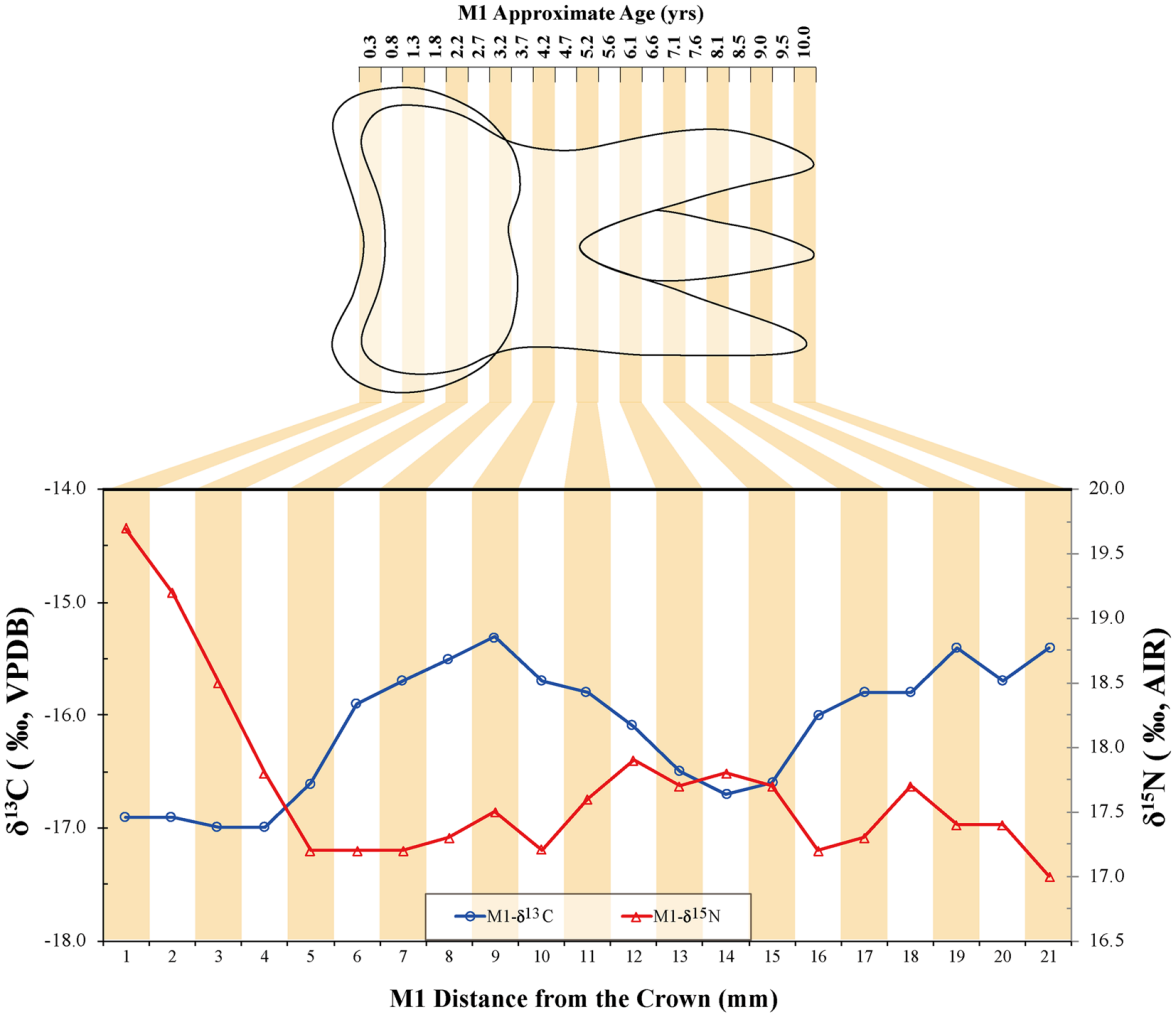
Stable carbon and nitrogen isotope results of incremental dentine from the M1 of Yingpan Man.

Yingpan Man’s first molar dentine serial section δ^13^C values range from − 17.0‰ to − 15.3‰ (mean ± SD = − 16.1 ± 0.6‰) (Figs. 3 and 4; Table S6). This indicates that his diet was a mixture of C_3_ and C_4_ dietary resources from birth to ~ 10 years old^24^. The δ^15^N results of the serial sections of Yingpan Man’s first molar range between 17.0‰ and 19.7‰ (mean ± SD = 17.7 ± 0.7‰). The first five sections of the M1 (closest to the crown) display the highest δ^15^N values which sequentially decrease by 2.5‰. This reflects the duration of breastfeeding and the weaning period for Yingpan Man. These δ^15^N results are exceptionally elevated, but are similar to the baseline plant and animal results from the Yingpan cemetery.

### Collagen, muscle and sequential hair sections of Yingpan Man

**Figure 5:**
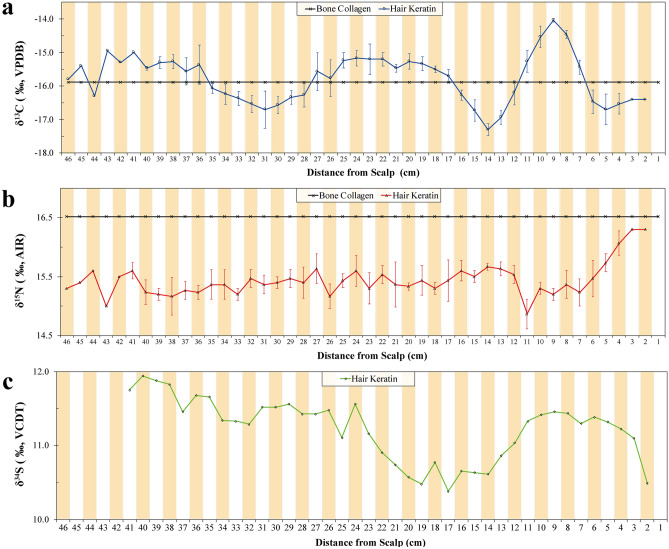
(**a**–**c**) Stable carbon, nitrogen and sulfur isotope values of bone and hair samples of Yingpan Man. (Note: Standard error of each hair section is labeled with a vertical error bar when sample duplication is over 2 times ($\ge 3)$).

Bone collagen from Yingpan Man’s patella yielded isotopic results of − 15.9‰ for δ^13^C and 16.5‰ for δ^15^N (Fig. 3; Tables S7, S8). While collagen from his mandible displayed isotopic results of − 13.7‰ for δ^13^C and 15.2‰ for δ^15^N. Two muscle samples collected from Yingpan Man’s knee exhibited δ^13^C values of − 16.7‰ and − 17.0‰ as well as δ^15^N values of 19.1‰ and 19.9‰ (Table S9 & S10). The δ^13^C results of the sequential hair sections display three cycles (~ 5 to 14, ~ 14 to 31, ~ 31 to 46 cm from the scalp, respectively) reflecting yearly seasonal changes in the consumption of C_3_ (e.g. wheat, barley) and C_4_ (millet) foods (range = − 17.3‰ to − 14.0‰; mean ± SD = − 15.8 ± 0.7‰; Fig. 5a; Table S11 & S12). The hair δ^15^N values show little variation from sections 12 to 46 cm (range = 15.0‰ to 16.3‰; mean ± SD = 15.4 ± 0.2‰; Fig. 5b; Tables S11, S12). These results indicate that Yingpan Man’s diet was relatively fixed in terms of trophic level.

However, the last 6–7 cm of hair closest to his scalp, which correspond his last ~ 6 months of life, display increasing δ^15^N results of ~ 1‰. This generally progressive ^15^N-enrichment in hair values before death implies a change in either nutritional status or a possible period of prolonged catabolic wasting before death^25^. Though the hair serial sections from 7 to 39 cm displayed δ^34^S values with relatively low variability (range = 10.4‰ to 11.9‰; mean ± SD = 11.2 ± 0.4‰; Fig. 5c; Tables S11, S12), the last 7 cm exhibits decreasing δ^34^S values of ~ 1‰ (range = 10.5‰ to 11.4‰; mean ± SD = 11.2 ± 0.3‰). This combined with the δ^15^N results indicates that the diet was terrestrial and relatively constant in terms of protein consumption during most of Yingpan Man’s lifetime, while dramatic changes occurred in the last ~ 6 months of his life.

## Discussion

### Environmental isotopic baseline of the Yingpan cemetery

Multiple body tissues of Yingpan Man, including his serial dentine, bone collagen, hair keratin and muscle, were isotopically analyzed. The δ^13^C measurements show a mix of C_3_ and C_4_ dietary inputs with values that range from − 17.3‰ to − 13.7‰, while the δ^15^N results are remarkably elevated and range from 14.9‰ to 19.9‰. According to the isotopic literature, plant δ^15^N values above 20‰ are relatively rare in terrestrial environments^22,26–28^. However, the δ^15^N values of the plants, animals and Yingpan Man are remarkably elevated (up to 23.3‰) compared to isotopic results from other parts of China and Europe (Fig. 3)^12^. This suggests that the entire ecosystem of this portion of Xinjiang is ^15^N-enriched. In addition, seeds of *Nitraria pamirica* having elevated δ^15^N results (up to 27.9‰) were found in the western region of the Pamir Plateau near the Afghanistan border^23^. Grains of wheat and barley from Shichengzi (ca. 202 BC–220 AD; ~ 400 km from Yingpan) yielded δ^15^N values that range from 14.6‰ to 19.8‰ (mean ± SD = 17.2 ± 1.5‰; n = 10) and 13.4‰ to 19.8‰ (mean ± SD = 17.3 ± 1.9‰; n = 12), respectively^29^. This evidence suggests that the entire Tarim Basin and greater Xinjiang has some of the most elevated terrestrial δ^15^N values in Eurasia. This is also supported by past isotopic work in China, which demonstrated a correlation between human δ^15^N values and annual mean precipitation, with individuals from Xinjiang having the highest δ^15^N values of all regions studied^12^. The cause of this ^15^N-enrichment must be at least partially environmental based on past isotopic studies^27,30^. The extreme aridity of the Yingpan cemetery site, which is located in the Taklamakan Desert, the driest region of China and characterized by little rainfall of 0 to 100 mm/yr and high evapotranspiration rates > 2500 mm/year (Fig. 1)^31^, results in a large plant ^15^N-enrichment by intensive evaporation of ^15^N-depleted ammonia (NH_4_^+^) from the soil. These elevated δ^15^N results are then translated up the food chain to the domestic animals and humans. This is compelling evidence that Yingpan man was born and raised in a ^15^N-enriched environment that was extremely arid, and that he consumed wheat/barley, broomcorn millet and grape that were grown locally.

### Breastfeeding, weaning and childhood dietary patterns of Yingpan Man

Isotopic analysis of dentine serial sections in human teeth permit an investigation of individual dietary patterns over the time period when the teeth were developing^24^. For Yingpan Man, his first molar (M1), represents the period of his life from birth to approximately 10 years old^32^. The first five 1 mm dentine sections from the M1 crown, corresponding to the first ~ 2.2 years of his life, show a steady decrease in δ^15^N of 2.5‰ (Fig. 4). This is evidence that Yingpan Man was fully weaned off breastmilk at or before the age of ~ 2.2 years old (Fig. 4)^33,34^, and is similar to findings of the Zhou Dynasty (1122–771 BC) sites of Boyangcheng^35^, Xiyasi and Changxinyuan^36^.

**Figure 6:**
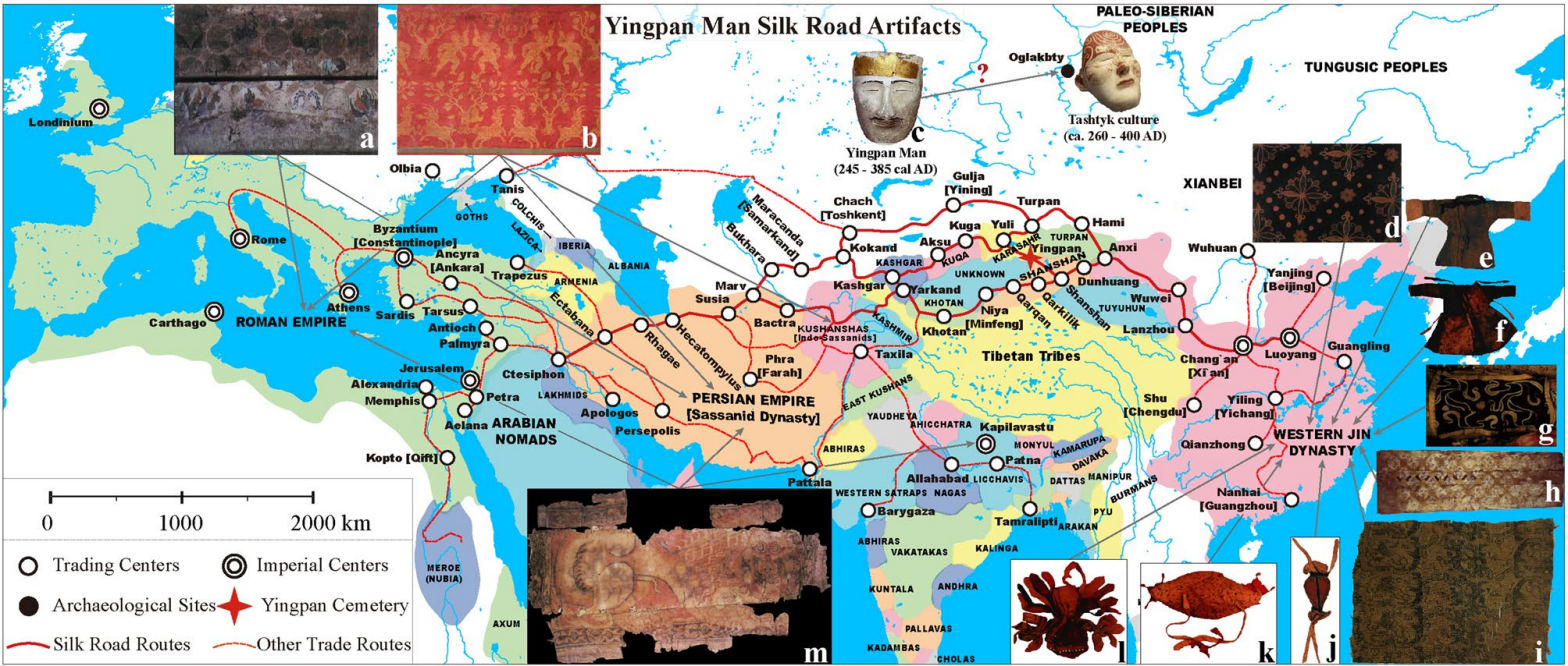
Yingpan Man’s grave goods mirroring the cultural contact across the Eurasian continent (~ 300 AD). **(a)** Decorative patterns of vase and flowers on the sideboards of Yingpan Man’s coffin. **(b)** Decorative patterns of nude putties, goats, bulls and pomegranate trees on Yingpan Man’s woolen caftan. **(c)** Yingpan Man’s white hemp mask with golden diadem (possibly related to the death masks from Tashtyk culture). **(d)** Woolen trousers with decorative patterns of double quatrefoil florals surrounded by lozenges made up of solid circles and flowers. **(e)** Miniature funerary summer coat. **(f)** Miniature funerary winter robe. **(g)** Embroidered armband. **(h)** Yellow painted wood coffin with decorative patterns. **(i)** A broken brocade that was decorated with Chinese characters of “Shou” and “You”. **(j)** Silk fish. **(k)** Crowing cockerel pillow. **(l)** Perfume sachet. **(m)** A tufted carpet with decorative patterns of a male lion. (Map was generated using GMT 5.2.1. The original pictures of artifacts were previously published^14^ and provided by Wenying Li. Pictures were modified using Adobe Photoshop CC 2015 V.1.2. The final layout was created in Adobe Illustrator CC 2019 V.23.1.1).

Archaeological evidence for infant feeding practices is rarely preserved in Xinjiang. However, two extraordinary feeding vessels (one made of goat breast skin, the other made from an ox horn) were previously discovered with the burial of a 10-month old infant mummy in the southern Tarim Basin^4,7^. This infant dates to ~ 1000 BC and was found at the Zhagunluke site which is ~ 460 km from the Yingpan cemetery (Fig. 1). In addition, historical documents also provide supporting evidence that children were fed with animal milk in ancient Xinjiang. For example, the Kharosthī scripts (written in a version of the north Indian Prakrit language) are a collection of contracts, letters and other documents (e.g. wood tablets, leather, silk, paper, etc.) that detail life, events, trading, taxes and agricultural practices during the third to fifth century AD of the Shanshan Kingdom (~ 250 km from Yingpan, see Fig. 6)^37^. It is recorded in these documents that a “milk fee” of cattle or camel was paid to the adoptee when children were adopted in ancient Shanshan, and this exchange was not only protected by the law but also needed to be ensured by witnesses^37,38^. Thus, there may have been a common tradition of feeding infants with milk from domestic animals (e.g. goat, cattle, camel, etc.) during the weaning process in ancient Xinjiang.

In contrast, the δ^13^C results of the first four dentine sections show little variability, which indicates that Yingpan Man consumed other foods/liquids from an early age and was not exclusively breastfed for a significant amount of time after birth (Fig. 4; Fig. S4)^34^. Between 4 and 9 mm, there is an increase in the δ^13^C values of 1.7‰, which corresponds to a late weaning and childhood diet from approximately 1.8 to 4.2 years old, where more millet was consumed (Fig. 4; Fig. S4). This millet possibly took the form of a gruel as there is little evidence that Yingpan Man consumed increased amounts of animal protein during this period. Similar isotopic patterns in dentine representing individuals that consumed increasing amounts of millet during weaning and early childhood were found at the Late Neolithic (4500 BP) Gaoshan site in Sichuan Province^39^. This suggests that millet may have had a long history of being used as a weaning or childhood food in China. From 9 to 14 mm, the dentine serial sections show a decline in δ^13^C values of 1.4‰ which indicates a period of increasing C_3_ foods in the diet from approximately 4.2 to 6.6 years old (Fig. 4; Fig. S4). Then from 14 to 21 mm the δ^13^C values increase again by 1.3‰, evidence of a change back to millet consumption from approximately 6.6 to 10 years old. Thus, over the first 10 years of life, Yingpan Man had frequent dietary shifts between C_3_-based and C_4_-based foods (Fig. 4; Fig. S4).

### Seasonal diet of Yingpan Man

In agreement with his dentine serial sections, the δ^13^C results of Yingpan Man’s hair display fluctuations between C_3_-based (e.g. wheat, barley) and C_4_-based (millet) foods (Fig. 5a). These hair δ^13^C results appear to follow a periodic trend, and suggest that his diet changed during different seasons of the year. In contrast, apart from the last 6–7 months of life, Yingpan Man’s hair δ^15^N values show little variation (Fig. 5b). However, a strong correlation was found between the variability of both the hair δ^13^C and δ^15^N values (Sperman’s r = − 0.534; sig = 0; n = 46; Fig. S5a), but not between the δ^13^C and δ^34^S values (Sperman’s r = 0.100; sig = 0.539; n = 40; Fig. 5; Fig. S5b). The lack of significant changes in the δ^15^N values is evidence that Yingpan Man’s protein consumption was relatively constant for approximately the last 4 years before death. This constant protein consumption, but variable intake of C_3_ and C_4_ plants, could suggest that in addition to directly consuming different amounts of wheat/barley and millet, Yingpan Man may have actually consumed domestic animals (goats, sheep, cattle) that were foddered on these crops at different times of the year. This possibility could explain the periodic variability in the δ^13^C values as well as the lack of variability in the δ^15^N values. In addition, the δ^34^S hair results also show little variation in the serial sections (~ 1.5‰). Yingpan Man’s δ^34^S results have a terrestrial range between 10.4‰ and 11.9‰^40–42^ and are similar to past δ^34^S values from the Proto-Shang site of Nancheng in Hebei Province^43^. While few δ^34^S studies have been reported for China for comparison, this lack of isotopic variability in sulfur suggests that Yingpan Man was likely not a Silk Road traveler, but stayed close to the local area during the last years of his life^42^. Future research involving strontium analysis on Yingpan Man’s hair serial sections and teeth will hopefully support or refute these findings^44^.

Regional evidence in support of seasonal dietary changes comes from apatite δ^13^C and δ^18^O results of serial sections of tooth enamel of domesticates from the pastoral sites of Dali, Begash and Tasbas in Kazakhstan (~ 750 km from Yingpan)^45^. Specifically, data from early and middle Bronze Age sheep, goats and cattle displayed periodic patterns in their δ^13^C_apa_ and δ^18^O_apa_ results that reflect the consumption of more millet during winter and more C_3_ plants during summer months, based on environmental inputs of body water^45^. A combination of radiocarbon dates and the application of the dietary mixing model (MixSIAR) identified early sheep and goats from this region (e.g. Dali, 2705–2545 cal BC) to be winter foddered with up to 44–50% of millet intake^45^. During later periods, this reliance on millet fodder during the winter months increased with some goats from Begash having 50–60% millet in their diets. Further, the relative contribution of millet to the diet of sheep and goats from the early phase (2345–2080 cal BC) of Begash reached up to 68–74% of the whole diet during winter months, especially from November to December^45^. This key study indicates that there is a long history and precedent for seasonal C_3_ and C_4_ feeding of domestic animals in Central Asia, and supports the isotopic findings of a seasonal diet in the hair of Yingpan Man.

In addition, isotopic analysis of sequential hair samples from mummies recovered from the Oglakhty cemetery in the Minusinsk Basin of southern Siberia, Russia (~ 900 km north of Yingpan) also show seasonal dietary variation with millet and fish consumed during the late summer and autumn^46^. These mummies of the Tashtyk culture date to the same period as Yingpan Man (third to fourth centuries AD), and interestingly were also buried with white painted gypsum funerary masks that are similar to the one that Yingpan Man was wearing^46,47^. This unique burial tradition could suggest links such as trade or an association between Yingpan Man and the Tashtyk culture and additional research is necessary to explore this possibility in more detail (Fig. 6).

Historical documents such as the Kharosthī scripts provide additional valuable information about seasonal diets and the foods consumed by the inhabitants of ancient Xinjiang^37^. These texts describe how the people of the Tarim Basin cultivated mainly wheat, millet and barley as their main cereal crops and that grapes were carefully managed for the production of wine^37^. Autumn (around the 10th month of the year), was mentioned as the time for harvesting crops and trading crops as well as wine and animals, and paying debts^37^. Thus, autumn would have been the time of the year with the most abundant amount of food resources, especially C_3_ foods like fruit, vegetables and wine. This is still true today, as the harvesting of agricultural products in the Tarim Basin mainly occurs in the month of October^48^. In contrast, during the winter months food resources would have been scarce with only non-perishable crops like millets and wheat available to guarantee the food supply “in the harsh winters of Inner Asia”^45^. Thus, more C_4_ foods were likely consumed during the winter and spring months while more C_3_ foods were consumed during the summer and autumn months.

If this information is applied to Yingpan Man’s hair δ^13^C results, it would suggest that the δ^13^C values decreased during the summer and autumn months (June to October) and likely reach a nadir during the middle of autumn or October. Therefore, Yingpan Man’s hair sections which are 8 to 10 cm, 18 to 25 cm and 36 to 43 cm from his scalp reflect the period of middle autumn (between August to October) while the hair sections which are 4 to 6 cm, 13 to 15 cm and 29 to 33 cm from his scalp represent late winter (December to February). This would suggest that Yingpan Man died ~ 4 months after the last drop in his δ^13^C hair values or in spring, possibly March or April (Fig. 5). In addition, the clothes in which Yingpan Man was buried, as well as his associated wardrobe, also provide information about the timing of his death^14,15^. The miniature robe placed on his chest was designed for winter, as it was long, double layered and the interior was lined with sheep’s wool (Fig. 2f). Whereas the miniature coat placed near his wrist was designed for summer as it was shorter and made only of a single layer of silk (Fig. 2p). Yingpan Man’s caftan, in which he was buried, was double layered with the outer layer made of wool and the inner lining made of silk, and this was likely designed either for the spring or autumn (Fig. 2k)^14^. Thus, Yingpan Man’s burial clothes combined with the isotopic and historical evidence indicate that Yingpan Man died sometime during the spring months^14,15^.

To better illustrate Yingpan Man’s seasonal dietary variation, two hair sections respectively representing the highest (Sample A: − 14.0 ± 0.1‰, 15.2 ± 0.1‰) and lowest δ^13^C values (Sample B: − 17.3 ± 0.2‰, 15.8 ± 0.1‰) were analyzed by a Bayesian mixing model with the application of FRUITS (Food Reconstruction Using Isotopic Transferred signals)^49,50^. As displayed in Fig. S6, isotopic data for millet, wheat/barley, grape and sheep/goat from the Yingpan cemetery were incorporated into the mixing model as feasible dietary sources for Yingpan Man in both scenarios (Sample A and Sample B). The isotopic fractionation between human hair and diet is corrected with an offset of 4.0 ± 0.5‰ for δ^13^C values^51–53^ and 4.5 ± 0.5‰ for δ^15^N values^51,54^. The relative contribution of different macronutrients is defined in the mixing model according to published records with 74 ± 4% of the carbon originating from protein and 26 ± 4% originating from carbohydrates and lipids and all of the nitrogen originating from protein^55,56^. The high hair δ^13^C value of − 14.0‰ (Sample A) represents a heavy reliance on millet consumption (39–67%; median = 53%) and a low amount of dietary input from wheat/barley (0–51%; median = 12%), grape (1–46%; median = 25%) and sheep/goat (0–30%; median = 4%), which likely reflects Yingpan Man’s diet during the winter and spring months (Fig. S6 and Table S13). In contrast, the low hair δ^13^C value of − 17.3‰ (Sample B) likely represents a summer/autumn diet with a decline in C_4_ foods, like millet (8–44%; median = 28%), as well as increased reliance on C_3_ foods like wheat/barley (1–71%; median = 21%) and sheep/goat (0–81%; median = 9%) (Fig. S6 and Table S13). However, the importance of sheep/goat and wheat/barley is likely under-estimated here given their small sample size and highly elevated δ^15^N values. Nonetheless, the FRUITS mixing model indicates that the varying consumption of plant foods, especially millet, is clearly responsible for the δ^13^C shifts of Yingpan Man’s diet.

### The last months of Yingpan Man

The hair δ^13^C results provide an estimate for the time of year Yingpan Man likely died. In addition, the hair δ^15^N and δ^34^S values can provide evidence for how Yingpan Man may have died. The last ~ 6 cm (closest to the scalp) of Yingpan Man’s hair show a general rise in δ^15^N by ~ 1‰ (Fig. 5b). This pattern is uncharacteristic compared to the other hair δ^15^N results that display little variation. This unique ^15^N-enrichment could represent a period of catabolic wasting due to the recycling of tissue proteins as a result of a prolonged illness^25,57,58^. Additional support for some sort of disease or period of illness comes from the fact that there is little change in the δ^13^C values but a slight decrease in the δ^34^S values during the last ~ 6 cm of Yingpan Man’s hair. Tissue catabolism is known to cause an increase in δ^15^N but little change in δ^13^C values in human hair^25^. Further, δ^34^S results are known to decrease in the red blood cells and serum (by ~ 1.5‰) of patients suffering from liver cancer^59^. As the last ~ 6 cm of hair displayed a slight decrease in δ^34^S by ~ 1‰, with the largest decline during the last month of life, this evidence in conjunction with the δ^13^C and δ^15^N values suggests that Yingpan Man did not die suddenly but likely suffered some type of debilitating sickness over the last months of his life before he succumbed. However, as most wasting diseases or illnesses leave no traces on human skeletons, it is difficult to define the specific disease that caused Yingpan Man’s death, and a detailed paleo-pathological study of Yingpan Man is needed in the future.

**Figure 7:**
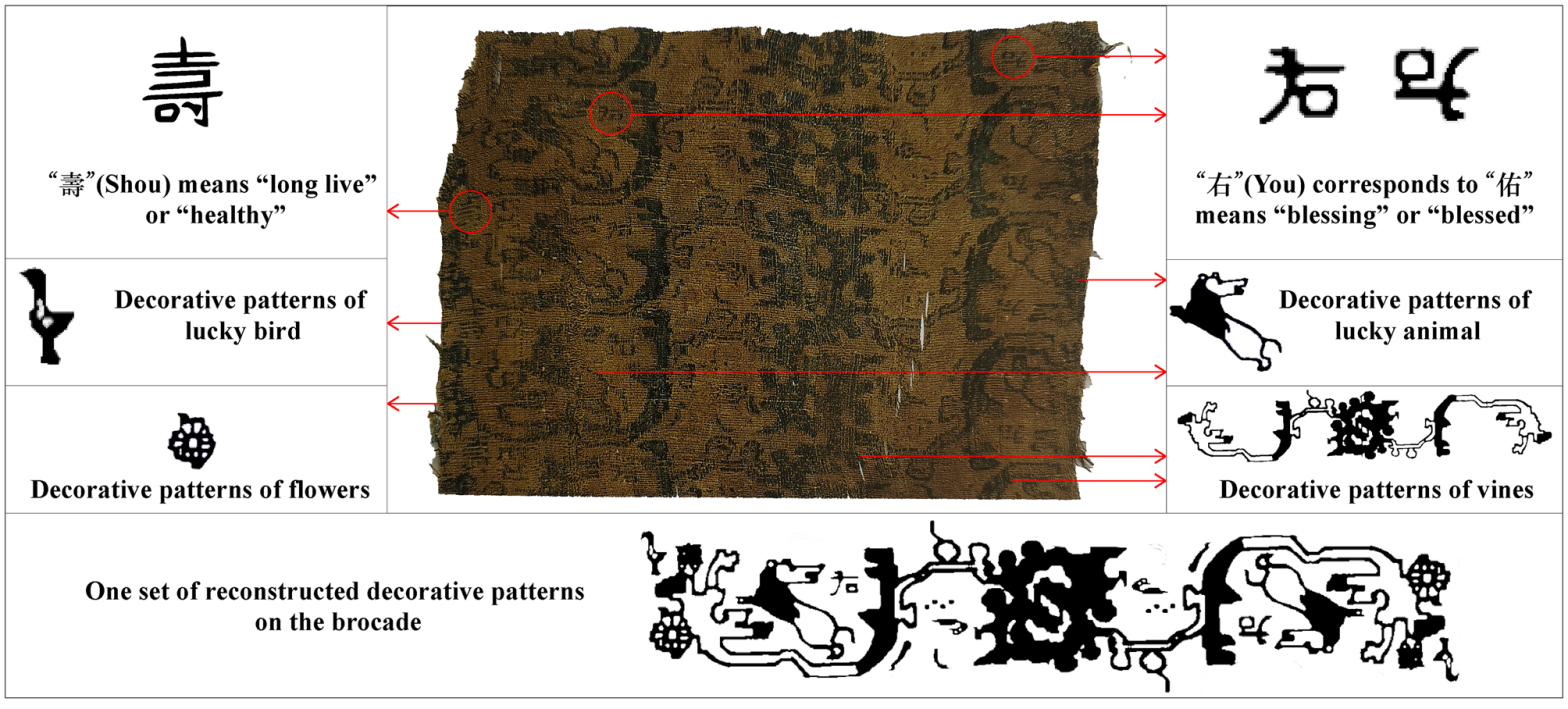
Photo and sketch showing details of the embroidered brocade that was found with Yingpan Man burial. (The original picture and sketch were previously published^14^ and provided by Wenying Li. Pictures were modified using Adobe Photoshop CC 2015 V.1.2. The final layout was created in Adobe Illustrator CC 2019 V.23.1.1).

The grave goods buried with Yingpan Man also suggest he may have suffered a compromised health status before death. In particular, a piece of tattered yellow brocade decorated with brown and blue images of vines, animals, birds, flowers, as well as the Chinese characters of “Shou” and “You” was found placed at a prominent position on the right side of Yingpan Man’s head (Fig. 7)^14^. According to Chinese historical literature sources, “You” means “blessing” or “blessed”. While, “Shou” means “long live” or “healthy” (Fig. 7)^60^. Similar grave goods with Chinese characters e.g. “Yan Nian Yi Shou Da Yi Zi Sun” (meaning “live longer and benefit the descendants”), “Chang Le Ming Guang Cheng Fu Shou You” (meaning “always be happy, be bright, be lucky and be blessed”), “Yong Chang” (meaning “always be prosperous”), were also frequently unearthed from contemporary and later cemeteries of the Tarim Basin, especially the nearby sites of the Loulan culture (third century BC to 448 AD, renamed as Shanshan in 77 BC, ~ 250 km from Yingpan), as grave goods that carry good wishes for the dead^15^. However, Yingpan Man’s brocade shows significant traces of wear or “rubbing” (Fig. 7). This is interesting as this brocade was not complete or new but well-worn to the point of being tattered and frayed, yet it was still placed at a very important position in Yingpan Man burial—just beside his head^14^. This suggests that this brocade may have been an important “lucky” health charm for Yingpan Man that was frequently used either by himself or by those who cared for him before burial. Thus, both isotopic and archaeological evidence suggest Yingpan Man suffered some type of illness during the last ~ 6 months of his life, likely in winter, and that he succumbed to this illness in the following spring.

### Who was Yingpan Man?

The social identity and status of Yingpan Man is enigmatic given the various cultural components of his grave goods and since the collection of his funerary objects are unique compared to all of the other burials found in the same cemetery^14^. This has created much controversy and debate about the social identity of Yingpan Man^7,14–16^. Thus, “Who was Yingpan Man?” and “Why was he buried here, in a normal unmarked grave with such lavish and exotic grave goods?” is an active topic of debate.

The physical anthropology of Yingpan Man was investigated but not formally published. According to which, the metric and nonmetric index of traits of his skeletal remains suggest a mix of both European and Mongolian features (Dong Wei, personal communication). However, facial reconstruction conducted by Dong Wei (personal communication) indicates that Yingpan Man’s facial structure is more characteristic of the features from Western Eurasia. This is consistent with the image of the face painted on his death mask and the fact that he wears a golden diadem across his forehead, which is more traditionally associated with Greece^4,7^. However, Yingpan Man’s white hemp mask is similar in style to the white painted gypsum masks of the Tashtyk culture from the Minusinsk Basin of Russia^46^. In addition, Yingpan Man and the mummies of the Oglakhty cemetery of Tashtyk date to nearly the same period (third to fourth centuries AD), and the polychrome silk cloth from the Tarim Basin has also been discovered in Oglakhty^46^. This evidence, while circumstantial, could suggest some form of association or that links with the Tashtyk may have taken place during the lifetime of Yingpan Man, possibly through trade or familial relationships (Fig. 6).

Other components of Yingpan Man’s burial provide important clues about his social identity and cultural affiliations in life^61^. The styles and types of grave goods of Yingpan Man display an unique mix of both Eastern and Western cultures and traditions that were likely common to inhabitants of Silk Road trading towns in Xinjiang during the third to fourth centuries AD (Fig. 6)^14–16^. However, there appears to be a strong eastern component in some of his funerary arrangements. For example, the burial practices associated with Yingpan Man: covering his face, filling his nose, burying his body fully clothed, covering it with a silken burial shroud, as well as using miniature funeral objects as grave goods are in accordance with the suggested burial rites of “Yan” (meaning “covering”), “Zhen” (meaning “filling”), “She Min Mu” (meaning “covering the eyes”), “Qin” (meaning “quilt” or “burial shroud”) mentioned in the Confucian literature of *Yili* (meaning “Rites”, formed during the Zhou Dynasties (1046 BC to 256 BC))^15,62^. In addition, the styles and designs of some of his grave goods are indicative of Chinese spiritual beliefs. For instance, the diamond and circle-shaped designs of decorative patterns on the cover and sideboards of Yingpan Man’s coffin are argued by some scholars to be the traditional “Lianbi” pattern (meaning “linked jades”) which symbolizes the jade burial suits (“Jinlü Yuyi”) that were popular in Han Dynasty burials of high status nobles in central and southern China (e.g. Nanyuewang tomb, Mancheng tomb)^15^. In particular, as jades are believed to be the ideal material for embalming in ancient China^63^, the “Lianbi” pattern on Yingpan Man’s coffin carries the symbolic meaning of preserving his body forever so that his soul and spirt will reach heaven (Fig. 6; Fig. S1)^15^.

The brocade “health charm” found to the right side of Yingpan Man also shows a clear affiliation with China, as it was decorated with the Chinese characters of “Shou” and “You”. This is important as some historical documents suggest that Kharosthī was the common language in the Tarim Basin at this time^37,38^, but Yingpan Man was likely more accustomed to Chinese spiritual beliefs according to this brocade (Figs. 6, 7). Moreover, the crowing cockerel pillow is also interesting as it was recorded in the Han Dynasty literature of *Lunheng* (meaning “*On Balance*”, compiled by Chong Wang during the Eastern Han Dynasty in 88 AD), that the dead “turns into ghosts” and “hurt the living ones”^64^, and that a crowing cockerel was believed to be able to expel evil spirts and ghosts^65^. Thus, a crowing cockerel pillow was frequently used in funerary practices in China since the Han Dynasty^66^, and it is still a common grave good today in some areas of modern China (e.g. Shandong and Guizhou Provinces)^65^.

**Figure 8:**
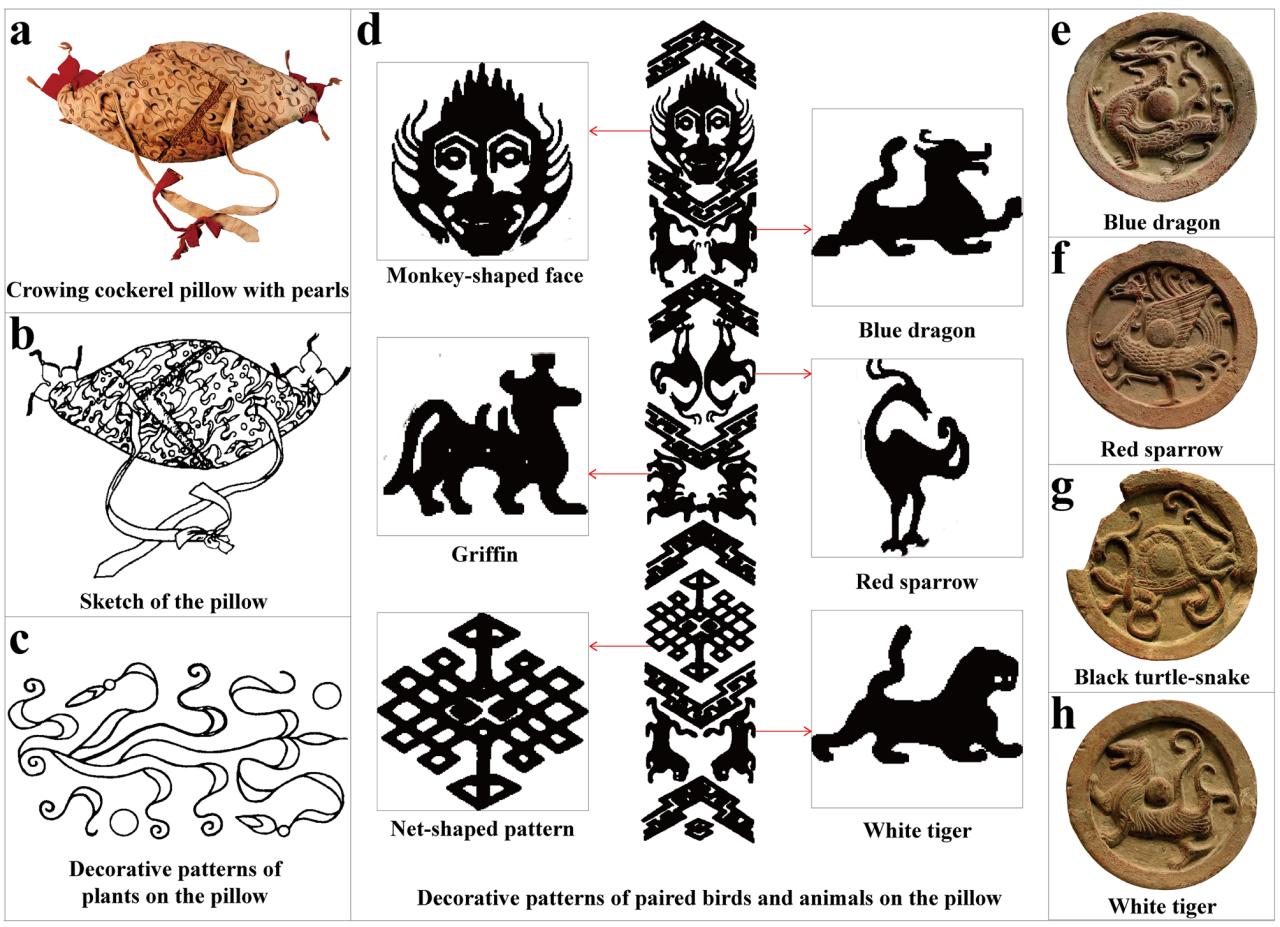
Crowing cockerel pillow. **(a**–**d)** Photo and sketch of the crowing cockerel pillow from Yingpan Man burial. **(e–h)** Decorative patterns of the four mythical beasts on Han Dynasty eaves tiles. (The original pictures and sketches were previously published^14^ and provided by Wenying Li. Pictures were modified using Adobe Photoshop CC 2015 V.1.2. The final layout was created in Adobe Illustrator CC 2019 V.23.1.1).

The eight decorative pearls attached to the crowing cockerel pillow are also important status markers regarding the identity of Yingpan Man (Fig. 8). In Xinjiang, pearls would have been long distance imported products from the coastal regions which are > 3000 km from the Yingpan cemetery. Ancient China, Egypt, Persia, Greece and India were known to have produced and prized pearls^67^. However, according to Chinese historic literature sources: *Shangshu* (meaning “the Book of Documents”; written by pre-Qin philosophers during the Zhou Dynasty ~ 1000 BC)^68^, *Hanshu* (meaning “the Book of Han”; written by Gu Ban during the Eastern Han Dynasty in 105 AD)^69^ and *Hou Hanshu* (meaning “the Book of Later Han”; written by Ye Fan during the Southern Dynasties from 432 to 445 AD)^70^, pearls were known as royal tributes in China since the Xia Dynasty (~ 2000 BC), and were mainly produced in the coastal cities of southern China, such as Panyu (modern Guangzhou City in Guangdong Province), Hepu (modern Hainan Province), Zhuya (modern Guangxi and Guangdong Province), etc.^67^ These coastal regions of China were connected to the Tarim Basin area via the Silk Road trading routes and were possibly the source for the pearls found on Yingpan Man’s pillow (Fig. 6). In particular, a silk pillow fully covered with unpolished natural pearls (placed beneath the tomb owner’s head, weighing ~ 470 g) and a lacquer box of pearls (weighing 4,117 g) were unearthed from the tomb of the King of the Nanyue Kingdom (a vassal state of Han) in modern Guangzhou^71^. This demonstrates a similar preference for pearls as decorations on pillows or as grave goods for high status individuals.

Moreover, the decorative patterns found on Yingpan Man’s crowing cockerel pillow are remarkable. These include the images of: a monkey-shaped face, a griffin-shaped beast and a net-shaped pattern as well as the images of the mythical beasts of the “blue dragon”, “red sparrow” and “white tiger” (Fig. 8d)^17,47,61,72^. Together with another mythical beast known as the “black turtle-snake”, these four mythical beasts were believed to be the guardians of the four cardinal directions and also represents four different colors and elements according to traditional Chinese cosmology (Fig. 8e–h). The dragon guarding the East and representing the color blue and the element of wood, the sparrow guarding the South and representing the color red as well as the element of fire, the tiger guarding the West and representing the color white and the element of metal, and the turtle-snake guarding the North and representing the color black and the element of water^62,73^. Thus, the images of these four mythical beasts were frequently integrated into the designs of cities, buildings, cemeteries and objects of ancient China for their symbolic function of protection^63^. In this context, the net-shaped pattern on Yingpan Man’s crowing cockerel pillow is likely a symbol of the four cardinal directions, while the monkey shaped face possibly symbolizes a human, that is to be protected. Notably, here, in the design of Yingpan Man’s crowing cockerel pillow, the image of the northern guardian of the turtle-snake is replaced by the Greek mythical beast known as Griffin (Fig. 8d)^16,47,74^. This change in imagery and symbolism could suggest that contact between the East and the West was via a northern route and that this was associated with some type of Greek influence or the Griffin. According to historical literature sources, a large number of Greeks migrated into Central Asia with Alexander the Great’s eastward expedition^3,5,6^. Hellenistic kingdoms such as Bactria (a.k.a. Daxia in the Chinese literature, located in the area of Pamir Plateau in modern Afghanistan, ~ 1900 km from Yingpan) subsequently became centers of Greek influence in this area and exported elements of Hellenistic culture to surrounding kingdoms that were located along the main routes of the Silk Road, such as Dayuan (located in the Fergana Valley, ~ 1300 km from Yingpan)^2,6,75^. Some ancient cities in the Tarim Basin area, such as Yingpan, Maideke and Yuansha, are also argued to have been influenced by Hellenistic elements as their city walls were circular in shape and this was clearly a unique architecture compared to the square-shaped traditional Chinese city walls^63,76^. It is highly probably that these Hellenistic elements were transported to the ancient city of Yingpan by the Silk Road trading routes which followed a north–south direction in this region, and therefore would support that Greek elements would be associated with the northern direction in this region of Xinjiang. In conclusion, Yingpan Man’s crowing cockerel pillow displays an unprecedented combination of Chinese spiritual beliefs incorporated with western motifs, and symbolizes the intertwining of Eastern and Western cultures in this part of Central Asia (Figs. 6, 8).

Even more significant uses of western components in Yingpan Man’s burial is also visualized in other grave goods, e.g. his: carpet, coffin, caftan. The decorative pattern of the male lion on Yingpan Man’s tufted woolen carpet is clearly an imported element as the lion was originally from Africa, Southern Europe, West Asia and India. Lions were not introduced into China until 87 AD, being sent to Emperor Zhang of the Eastern Han Dynasty as a gift from Pacorus II, the King of the Anxi Empire (a.k.a. Parthian Empire (247 BC to 224 AD, replaced by the Persian in 226 AD, located in modern Iran))^70^. In particular, this exquisite lion-decorated carpet suggests a very high social status for Yingpan Man as lions were deified in ancient China since the Han Dynasty and were used as symbols of power, authority and royalty as it was in other areas of the Eurasia (Fig. 2a)^15,61^. Moreover, it is also argued by some scholars that the motif of the lion on this carpet is from the Buddhist art of India, as lions are frequently mentioned in Buddhist stories and a Buddhist temple was also found at the site of Yingpan (Fig. 6)^17,77^.

**Figure 9:**
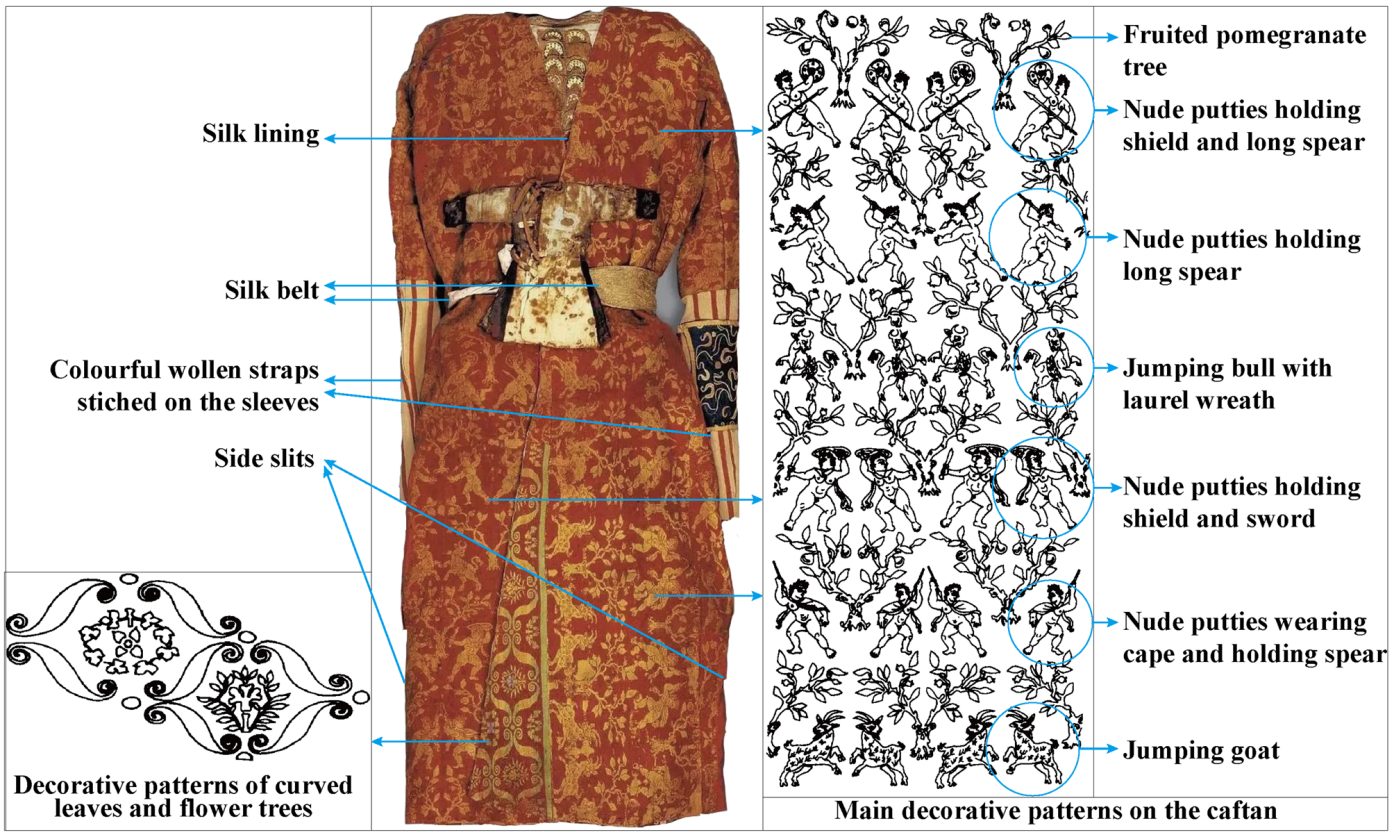
Photo and sketch of the decorative patterns on Yingpan Man’s caftan. (The original pictures and sketches were previously published^14^ and provided by Wenying Li. Pictures were modified using Adobe Photoshop CC 2015 V.1.2. The final layout was created in Adobe Illustrator CC 2019 V.23.1.1).

In addition, though the main decorative patterns on Yingpan Man’s coffin are the “Lianbi” pattern, images of vines, leaves, pomegranate flowers and vases were also depicted inside of the diamond patterns (Fig. S1)^15^. Among which, the image of pomegranate flowers is clearly an imported element from the West as pomegranates were originally domesticated in the middle East ~ 5000 years ago and were not introduced into China until the Han to Jin Dynasties, or with the flow of goods along the Silk Road^78^. In particular, the pomegranate was used as a symbol of health, fertility and rebirth as mentioned in many ancient cultures, especially in Greek and Turkish myths^78^. Thus, this is particularly interesting as the image of pomegranate appears not only on Yingpan Man’s coffin as flowers on the headboard and footboard (Fig. 6; Fig. S1), but also on his caftan as decorative patterns of fruited-trees (Figs. 6, 9). Specifically, the decorative patterns on Yingpan Man’s woolen caftan consists of six sets of nude puttis and animals with fruited pomegranate trees standing in between (Fig. 9)^14^. In particular, each of these six sets of images is composed of a symmetrical pair of confronting muscular puttis or animals (goats or bulls) that are either leaning away from or toward each other^14^. The nude puttis are holding either a spear, sword or a shield with capes swirling from their shoulders, while the animals of goats and bulls are in the pose of jumping and the bulls have laurel wreaths around their waists (Fig. 9)^14^. A similar design of stance and composition of figures was also discovered in a mosaic floor from the Villa of Good Fortune at Olynthos, Greece (paired female figures with weapons, fourth century BC)^79^ as well as another mosaic floor from Pella, the Macedonian capital (two nude youths with capes flying on their shoulder and weapons held in their hand, about to attack an animal in between of them, 325 to 300 BC)^79^. Thus, it is suggested that this caftan was the work of a weaver familiar with both Western and Eastern motifs as the character and poses of the nude puttis are clearly Western in style and appearance, the fruited pomegranate tree is believed to be a Persian motif, while the paired facing animals of goats and bulls are similar to the animal art of Central Asia^8^. However, this caftan was completed using the technique of double-weaving, the lining and belt were made of silk^14^, while the design of the side slits are indicative of a localized adaptation for horse-riding^15^. Moreover, analysis using high performance liquid chromatography (HPLC) on the red and yellow threads of this caftan suggest that they were dyed in a local workshop with indigenous materials of *Rubia Tinctorum* and *Populus Pruinosa Schrenk*, respectively^80,81^. In conclusion, the design and style of Yingpan Man’s caftan is unprecedented, and it is a masterpiece that combines both Greek/Roman, Persian, Central Asian, Chinese and local elements (Fig. 6).

The opulence and fine quality of the objects buried with Yingpan Man indicate that he must have had a high social status before death^14,47^. Given the importance of the town of Yingpan as a trading center on the Silk Road, the excavators who discovered Yingpan Man suggested that he was a wealthy merchant from the West^14^. Others have proposed that Yingpan Man might have been a Sogdian merchant since the Sogdians (an Iranian-speaking people whose homeland lay near Samarkand in what is now Uzbekistan) were the richest traders along the route^7^. However, given the relatively young age of Yingpan Man before his death (~ 30 years old), it is unlikely that he amassed all his fortune and high social status only through trade, and possibly by inheritance or military feats. The government of the Jin Dynasty established the administrative organization of “Xiyu Zhangshi Fu” (meaning “Chief Governor of the Western Regions”) in ancient Xinjiang, and the capital city of “Xiyu Zhangshi Fu” was located nearby Lop Nur and is very close to the ancient city of Yingpan (~ 185 km away)^15,16^. In addition, comparison of Yingpan Man’s burial to other contemporary burials from Gansu also suggest that Yingpan Man was possibly a military official from the government of Central China^16^. More supporting evidence comes from the embroidered armband that was buried with Yingpan Man as colorful armbands were suggested to be used by soldiers for the protection from evil forces in ancient Xinjiang (Fig. 6)^15^. An additional explanation is that Yingpan Man was a noble or even a king of the nearby state named Shan (a.k.a. Moshan)^76^, and the ancient city of Yingpan was suggested to be the capital city of this state^15^. An alternative explanation is that Yingpan Man belonged to a local noble family who were displaced from Bactria to the southern Tarim Basin after civil strife in the Kushan Empire at the end of the second century AD, given the popularity of Kushan arts in this area during the Han to Jin Dynasties^82,83^. The isotopic evidence presented here, in particular the hair δ^34^S results, add additional information to Yingpan Man’s identity. The lack of δ^34^S isotopic variability (10.4‰ to 11.9‰) over the last ~ 3–4 years of life indicates that Yingpan Man was not a Silk Road traveler or merchant, at least during this period of his life. Thus, Yingpan Man appears to have been a local, possibly a governmental official or royal to this region of the Tarim Basin, perhaps from the nearby state of Shan. This might suggest why he was buried in the Yingpan cemetery as it was purported to be capital of this ancient state.

## Conclusion

**Figure 10:**
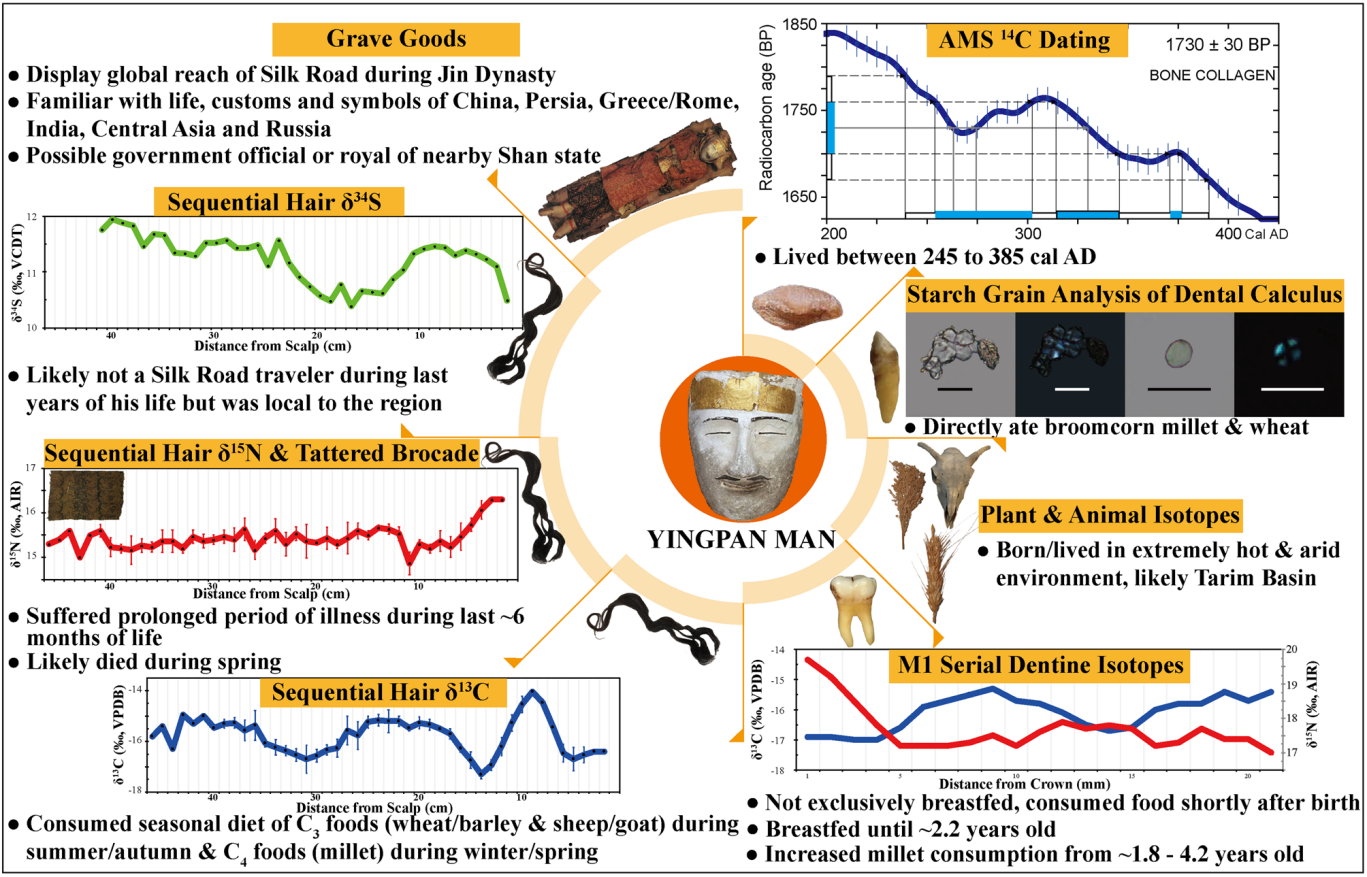
Graphic conclusions summarizing the findings of this work. (The original pictures of artefacts were previously published^14^ and provided by Wenying Li. The photos of plants were previously published^20^ and provided by Hongen Jiang. Pictures of analyzed human bone, teeth, hair and animal skull were photographed by Tingting Wang. Pictures were modified using Adobe Photoshop CC 2015 V.1.2. The final layout was created in Adobe Illustrator CC 2019 V.23.1.1).

The work presented here is summarized in Fig. 10 and provides a wealth of new information about Yingpan Man and his relation to the Silk Road. A detailed review of his physical anthropology and funerary materials found that he shared or must have been familiar with many aspects of life, customs and symbols associated with different parts of Eurasia, including: China, Persia, Greece/Rome, India, Central Asia and possibly the Tashtyk of the Minusinsk Basin of Russia. This directly demonstrates the enormous global influence and the extent of the flow of goods, ideas, and people were having on the populations of the Silk Road settlements in Central Asia and likely all across the Silk Road trading routes from East to West and North to South during the third to fourth centuries AD. However, there is still much additional research needed, including detailed paleopathological and ancient DNA analysis as well as additional isotopic measurements, and these are topics for future research. It is hoped that this future work will reveal even more lost secrets about this most enigmatic and amazing mummy of Xinjiang: Yingpan Man.

## Materials and methods

### Radiocarbon dating

A bone sample from the patella of Yingpan Man was radiocarbon dated by AMS at Beta Analytic Inc., Miami, Florida, USA. The date was calibrated using CALIB REV 7.1.0 with the IntCal 13 calibration curve and the result is displayed in Table S2^84^.

### Starch grain analysis

Starch grain analysis was applied to the dental calculus of Yingpan Man’s canine to investigate the foods, especially plant foods, that were directly ingested before death^85,86^. Modified based on the non-destructive protocol outlined by Piperno and Dillehay^87^, dental calculus was sampled from Yingpan Man’s canine using a dental scalpel to remove small flakes into a round bottomed 5 mL centrifuge tube. Calculus was ground into smaller particles with a glass bar, and then rinsed in distilled water and placed into an ultra-sonic bath for 30 min at room temperature to deflocculate the calculus and ease dispersal. Finally, samples were centrifuged 3 times (3000 rmp for 10 min each time), and the supernatant was pipetted off until less than 0.5 mL was left for microscopic observation. The remaining sample was mounted on a microscope slide in a 1:2 glycerin/water solution^87^, and examined under a compound light microscope equipped with a set of polarizing lenses (Nikon Eclipse LV100POL) at × 200 magnification. Starch grains were observed, measured and photographed at × 500 magnification (Fig. S2). They were then compared to a reference collection of starch grains from modern plants and published documents^18,19,21^ to identify to family, genus or species level where possible.

### Stable isotope ratio analysis

#### Pre-treatment of plant remains

A total of 14 plant samples from other burials of the Yingpan cemetery were collected and measured in order to establish an environmental baseline for the diet of Yingpan Man. These samples represent a variety of plant species and include both C_3_ cereals (e.g. wheat (*Triticum aestivum*)/barley (*Hordeum vulgar*): stalks (n = 2)), C_4_ cereals (e.g. broomcorn millet (*Panicum miliaceum*): stalk (n = 2), caryopsis (n = 1), palea (n = 1), lemmas (n = 1) & spikes (n = 1)), C_3_ wild plants (*Sophora alopecuroides*: leaves (n = 2), stalks (n = 2)), C_4_ wild plants (*Leymus secalinus*: leaves (n = 1)) and fruit (grape (*Vitis vinifera*): pulp (n = 1)). The grains of the broomcorn millet were too small to be individually analyzed so five grains were combined and used for a single isotopic measurement. Details are presented in Table S3. Since these desiccated plant samples were extremely well preserved and not charred, a relatively simple protocol for preparation (cleaning with distilled water and then freeze drying) was selected to minimize the influence of chemical solutions on these plants^88^.

#### Collagen extraction

Collagen was extracted from ~ 1 g of bone from the right patella and mandible of Yingpan Man at the Key Laboratory of Vertebrate Evolution and Human Origins of Chinese Academy of Sciences, Institute of Vertebrate Paleontology and Paleoanthropology, Chinese Academy of Sciences in Beijing, following the protocol outlined in Richards & Hedges (1999)^89^, with the addition of ultrafiltration prior to lyophilisation^90^. In addition, to create an isotopic baseline for the diet of Yingpan Man, collagen was extracted from the cranium of a goat (from 99BYYM7, see Fig. S3f) and a rib sample from a sheep (from 99BYYM8, see Fig. S3e) that were recovered from different burials in the Yingpan cemetery, following the same protocol as above.

#### Muscle pre-treatment

Two sections of desiccated muscle was collected from the knee of Yingpan Man to investigate his diet over a relatively short time period (2 ~ 3 months) before his death^91^. Modified based on the protocols outlined in White et al.^92^ and Finucane^93^, the muscle sample was first rinsed with methanol in order to remove the surficial contaminations. The sample was then defatted by ultrasonication in a 2:1 methanol: chloroform solution for 30 min. The chloroform methanol solution was changed every 24 h until all lipids were removed. The sample was air dried in a room temperature oven after having been washed three times with distilled deionized water. Additionally, one piece of desiccated sheep muscle from a different burial (from 99BYYM8, see Fig. S3e) of the Yingpan cemetery was also sampled and prepared following the sample protocol as above to provide a comparison for the dietary reconstruction of Yingpan Man.

#### Hair preparation

Three bundles of hair were collected from Yingpan Man with care taken to maintain the original orientation and alignment. ~ 20 hair strands were sampled from each bundle in order to represent a full range of Yingpan Man’s hair in both active and inactive phases (Fig. S7)^94^. To correspond with its average monthly growth rate, the hair samples were sequentially sectioned into 1 cm segments. With a total length of 39 cm, 43 cm and 44 cm respectively, the three bundles of hair have the potential to record the dietary and nutritional information of Yingpan Man for approximately 3–4 years of time before his death. Following the protocols outlined by Fuller et al.^95^ the sequential hair samples were then soaked in methanol to remove contaminations. After being ultrasonicated 3 times in a 2:1 methanol:chloroform solution (30 min each time, replaced with new chloroform methanol solution each time), the hair samples were washed with distilled water. Finally, the hair samples were freeze dried and homogenized with a mortar and pestle.

#### Dentine serial sections

To investigate the breastfeeding and weaning practices of Yingpan Man and his diet during early childhood, 21 serial sections of dentinal collagen from his first molar (M1) were sampled and extracted following “Method 2” mentioned in Beaumont et al.^96^ with the modification of demineralizing the teeth at room temperature. The age of each serial dentine section was assigned according to the age upon which different parts of the tooth initiates and complete formation^32^. Specifically, the M1 generally initiates formation just prior to birth and the entire tooth completed formation at ~ 10 years old^32^. Thus, serial sections of the dentinal collagen from the M1 of Yingpan Man covers most of his lifetime from birth to ~ 10 years old.

#### Measurement of stable isotope ratios

All pretreated tissue samples were measured in duplicate or triplicate in the Archaeological Stable Isotope Laboratory (ASIL), the Department of Archaeology and Anthropology at the University of the Chinese Academy of Sciences. Specimens were placed into tin capsules with ~ 3 g of plant and ~ 0.5 mg of collagen, keratin and muscle samples for measurement of δ^13^C and δ^15^N values. As for the determination of δ^34^S values, approximately 1.2 mg of hair keratin samples were loaded into tin capsules with additional WO_3_ (~ 0.5 mg) to improve combustion. The mass spectrometer was an IsoPrime 100 IRMS coupled with the Vario PYRO cube. The stable isotope results were analyzed as the ratio of the heavier isotope to the lighter isotope (^13^C/^12^C or ^15^N/^14^N or ^34^S/^32^S) and reported as “δ” in parts “per mil (‰) relative to internationally defined standards for carbon (Vienna Pee Dee Belemnite, VPDB), nitrogen (Ambient Inhalable Reservoir, AIR) and sulfur isotopes (Vienna Canyon Diablo Troilite, VCDT)^97^. In addition, sulfanilamide was used as a reference material for elemental analysis, IAEA-600, IEAE-N-1, IAEA-N-2, IAEA-CH-6, USGS-40, USGS-41, USGS-42, USGS-43, IAEA-S-2 and IAEA-S-3 were used as standards for stable isotope ratio analysis. Among which, USGS-40 and USGS-41 were used as standards for two-point calibration of δ^13^C and δ^15^N measurements, IAEA-S-2, IAEA-S-3 and USGS-43 were used as standards for calibration of δ^34^S values. Moreover, for every 10 samples, a collagen lab standard (δ^13^C value of 14.7 ± 0.2‰ and δ^15^N value of 6.9 ± 0.2‰) was also inserted in the run for data monitoring. Based on the SD of the inserted calibration standards, the measurement errors were less than ± 0.2‰ for δ^13^C and δ^15^N, and less than ± 1.0‰ for δ^34^S. The measured isotopic results are all listed in the Supplementary Materials with plant samples presented in Table S3 (summarized) and Table S4 (detailed), animal samples in Table S5, serial dentine of M1 in Table S6, human bone and canine collagen in Table S7 (summarized) and Table S8 (duplicated), human muscle in Table S9 (summarized) and Table S10 (detailed), human hair in Table S11 (summarized) and Table S12 (detailed).

#### Ethics declarations

Permission was obtained from appropriate authorities from where the samples were collected and from where the study was carried out. The involved authorities are detailed in the affiliation list of this manuscript with corresponding co-authors representing each affiliation. In addition, this research uses archaeological plant, animal and human samples. Informed consent of using, analysing and publishing these samples (as well as their data) was obtained from the Institute of Archaeology and Cultural Relics of Xinjiang (where the samples were collected). All activities involved in this research are well-complied with the ethical principles, applicable international and national laws as well as the relevant guidelines and regulations.

## Supplementary Information

Supplementary Information.

## Data Availability

All data needed to evaluate the conclusions in the paper are presented in the paper and/or the Supplementary Materials. Additional data related to this paper may be requested from the author.
