# Supplementary material for: Revealing lost secrets about Yingpan Man and the Silk Road
Source: Sci Rep. 2022 Jan 13;12:669. doi: 10.1038/s41598-021-04383-5 (PMC8758759; doi:10.1038/s41598-021-04383-5)
Supplement: Supplementary file 1 — Supplementary Information. [file 41598_2021_4383_MOESM1_ESM.pdf]

Supplementary Materials for *Revealing lost secrets about Yingpan Man and the Silk Road* by Tingting Wang, Benjamin. T. Fuller, Hongen Jiang, Wenying Li, Dong Wei and Yaowu Hu.

Supplementary Figures:

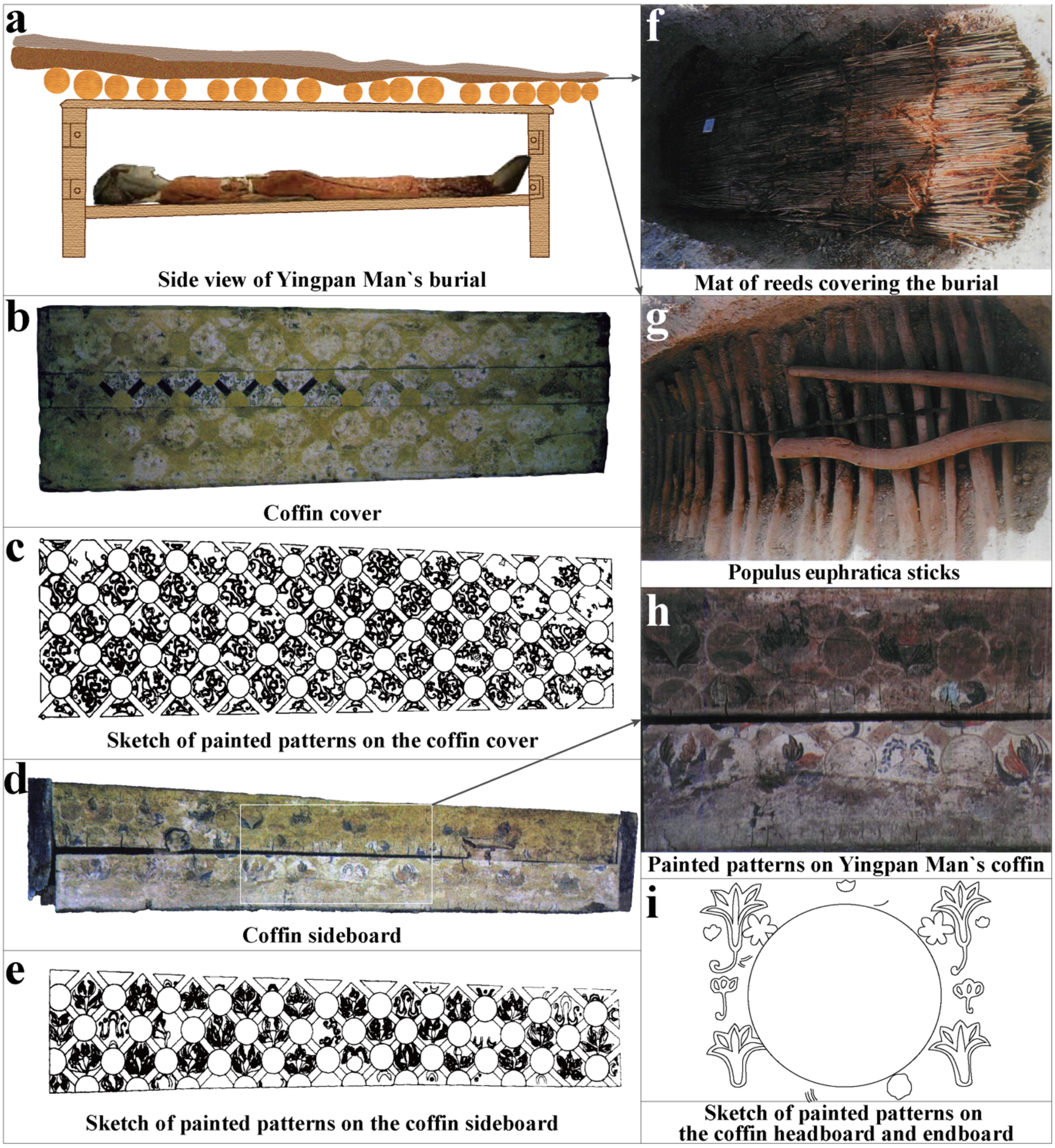

Fig.S1 Yingpan Man Burial. (a) Side view of Yingpan Man`s burial. **(b)** Photo of the coffin cover. **(c)** Sketch of painted patterns on the coffin cover. **(d)** Photo of the coffin sideboard. **(e)** Sketch of painted patterns on the coffin sideboard. **(f)** Mat of reeds covering the burial. **(g)** Photo of Populus euphratica sticks covering Yingpan Man`s coffin. **(h)** Decorative patterns of vase and flowers on the coffin sideboard. **(i)** Sketch of painted patterns on the coffin headboard and endboard. (The original pictures were previously published[1] and provided by Wenying Li. Pictures were modified using Adobe Photoshop CC 2015 V.1.2. The final layout was created in Adobe Illustrator CC 2019 V.23.1.1)

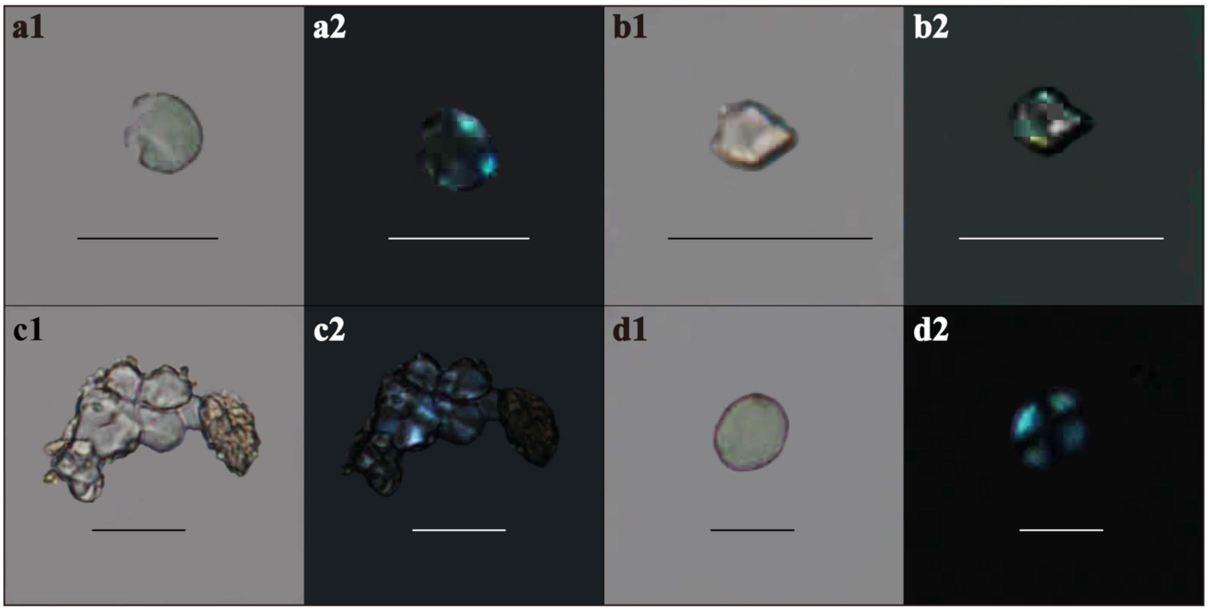

Fig.S2 Images of starch grains recovered from the dental calculus of Yingpan Man`s canine. (**a1-a2**. Destroyed starch grains that are unidentifiable; **b1–b2**, **c1-c2**. Starch grains that are most likely from the family of Poaceae, possibly millets; **d1-d2**. Starch grains consistent with some members of the Triticeae, possibly wheat; a1, b1, c1 & d1: transmitted light, 500×, scale bars = 20 μm; a2, b2, c2 & d2: cross-polarized light, 500×, scale bars = 20 μm).

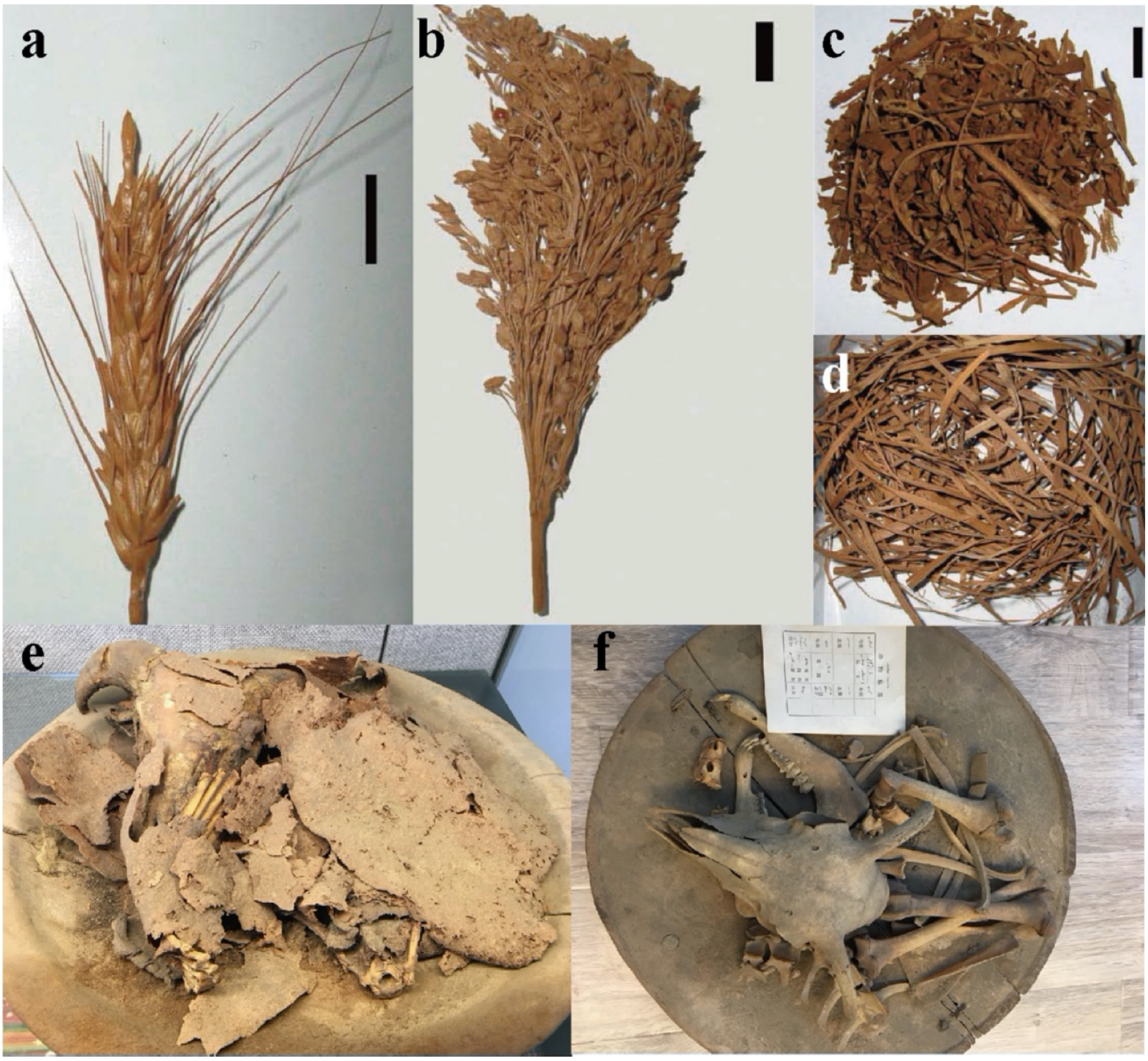

Fig.S3 Plant, animal and food samples from Yingpan cemetery. (a) Well preserved panicle of wheat from Yingpan cemetery (95BYYM19). **(b)** Front view of compact panicle of broomcorn millet unearthed from Yingpan cemetery (95BYYM26). **(c)** Leaves of *Sophora alopecuroides* from Yingpan cemetery (95BYYM20). **(d)** Leaves of *Leymus hochst* unearthed from Yingpan cemetery (95BYYM66). **(e)** Remains of sheep and pancake found in a wooden plate in Yingpan cemetery. **(f)** Remains of goat placed in a wooden plate in Yingpan cemetery. (Pictures of (a) – (d) were previously published and provided by Hongen Jiang[2]. Pictures of (e) & (f) were photographed by Tingting Wang. The final layout was created in Adobe Illustrator CC 2019 V.23.1.1)

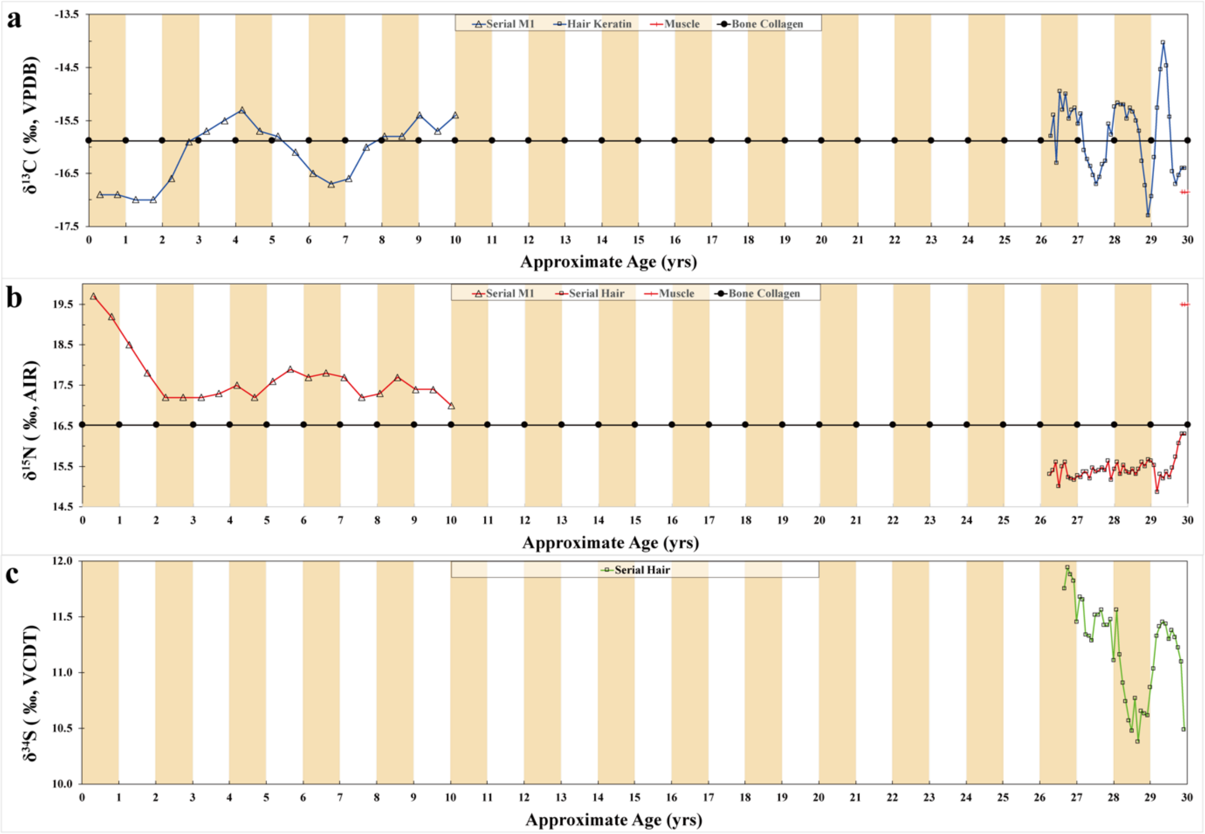

Fig.S4 (a), (b) & (c) Stable carbon, nitrogen, sulfur isotopic life history of Yingpan Man.

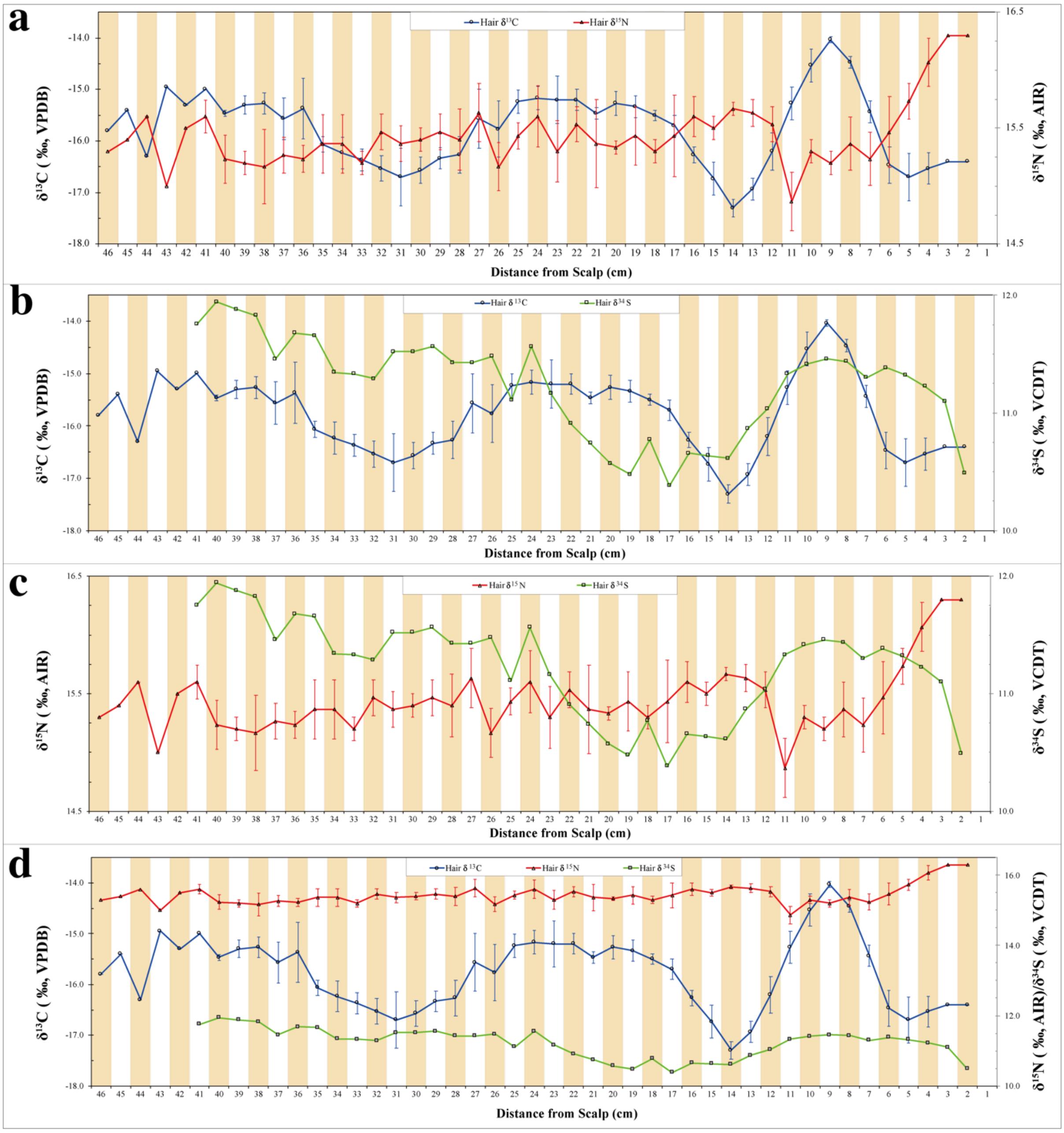

Fig.S5 (a), (b), (c) & (d) Comparison between the isotopic values of $\delta^{13}C$ vs. $\delta^{15}N$, $\delta^{13}C$ vs. $\delta^{34}S$, $\delta^{15}N$ vs. $\delta^{34}S$ and $\delta^{13}C$ vs. $\delta^{15}N/\delta^{34}S$ of hair samples from Yingpan Man. (Note: Standard error of each hair section is labeled with a vertical error bar when sample duplication is over 2 times (≥ 3)).

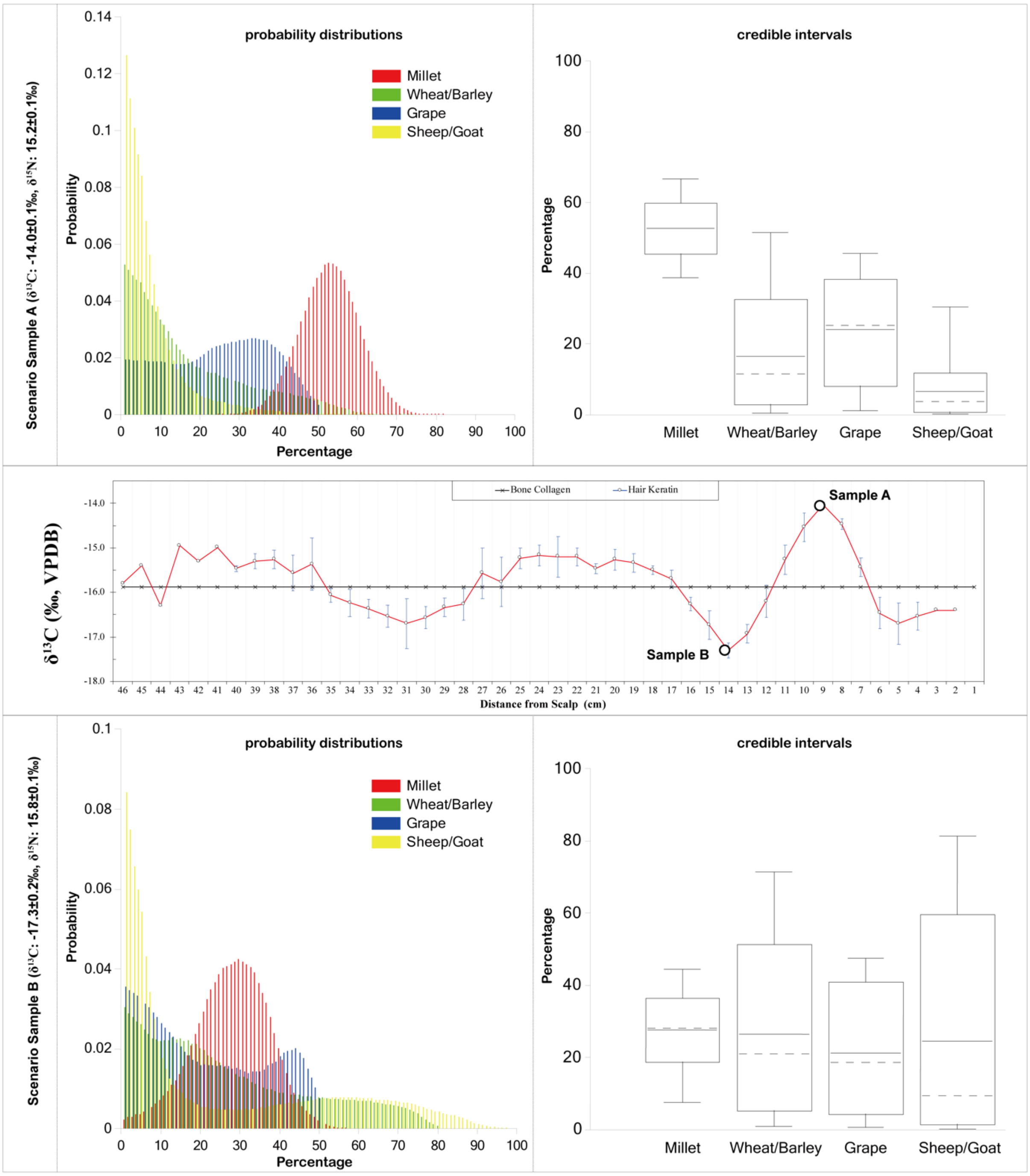

Fig.S6 Results of Bayesian Mixing Model with FRUITS. (Note: for the credible intervals of the mixing model, boxes represent a 68% credible interval (corresponding to the 16th and 84th percentiles) while the whiskers represent a 95% credible interval (corresponding to the 2.5th and 97.5th percentiles); the horizontal line represents the estimated mean while the horizontal discontinuous line represents the estimated median (50th percentile)).

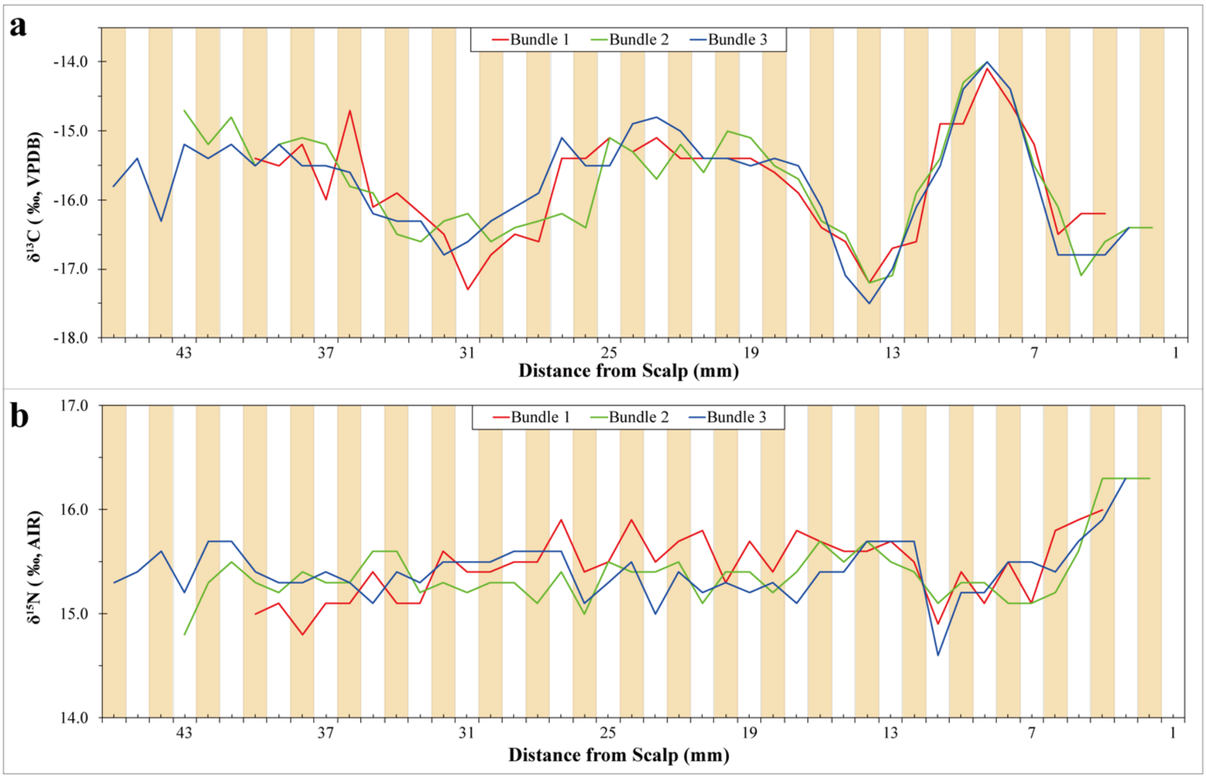

Fig.S7 (a) & (b) Comparison of stable carbon and nitrogen isotope results of all measured hair samples (3 bundles) from Yingpan Man.

Supplementary Tables:

Table S1. List of artefacts that are found in Yingpan Man`s burial.

Name	Artefact No.	Technique	Shape & Style	Size (cm)	Color	Material	Patterns & Decorations	Position	Figure
Wooden coffin	-	Mortise and tenon joint structure	Rectangle;	L=216, W=58~70, D=68~72;	White, red-brown, red-orange, red, green, yellow, blue, black;	Wood;	"Lianbi pattern" (circles and diamonds); curved grasses; flowers, vases, leaves; vines; pomegranate flowers;		Fig.2 b; Fig.6 a, h; Fig.S1;
Lion-decorated tufted carpet	M15:1	Plain weave; tufted;	Rectangle;	312*178	Bright red, dark yellow, medium yellow, orange, green, grass green, navy blue, acid blue, light blue, pink, dark brown, white;	Wool;	A male lion; geometric patterns;	Draped on the top of the coffin;	Fig.2 a; Fig.6 m;
Silk burial shroud	M15:2	Plain weave;	Rectangle;	181*74	Light yellow;	Silk (*"Juan"*)	-	Body cover;	Fig.2 c;
Woolen caftan	M15:3	Double weave; hand sewing;	Overlapped collar; right lapel;	110*185	Red, yellow, green, blue;	Wool;	Nude puttis; animals (goats and oxen); pomegranate trees;	Outfit;	Fig.2 g, k; Fig.6 b; Fig.9;
Woolen trousers	M15:4	Plain weave; lockstitch;	-	115	Light yellow, dark blue, red, pink-green;	Wool;	Double quatrefoil floral patterns surrounded by lozenges made up of solid circles and flowers;	-	Fig.2 h, i, k; Fig.6 d;

Crowing cockerel pillow	M15:5	Plain weave; lockstitch;	-	46*8	Light yellow, red, dark blue, brown, grass-green, off-white, light blue, grey, brown-red;	Silk (“*Qi & Juan”*), pearls;	Curved grasses; monkey-shaped face; blue dragon; red sparrow; net-shaped pattern; white tiger; griffin;	Beneath Yingpan Man`s head;	Fig.2 m; Fig.6 k; Fig.8;
White hemp mask with a golden diadem	M15:6	Lacquer-making;	-	23.4*19.8*11.7	White, black;	Hemp (“*Ma*”), gold;	Human face;	Covered on Yingpan Man`s face;	Fig.2 d, j, k; Fig.6 c;
Miniature funerary winter robe	M15:7	Plain weave; hand sewing;	Round neck; right lapel; with belt;	22.2*11.4	Pale blue, light yellow;	Silk (“*Juan*”), wool;	-	Placed on Yingpan Man`s breast;	Fig.2 f, k; Fig.6 f;
Miniature funerary summer coat	M15:8	Plain weave; hand sewing;	Overlapped collar; right lapel; with belt;	26.8*44.9	Yellow, light yellow;	Silk (“*Juan*”);	-	Nearby Yingpan Man`s left wrist;	Fig.2 k, p; Fig.6 e;
Embroidered armband	M15:9	Compacted plain weave; lockstitch; hand sewing;	Rectangle;	14*8	Blue, yellowish brown, brown, dark green;	Silk (“*Jian*”);	Flowers; vines;	Tied on Yingpan Man`s left arm;	Fig.2 k, o; Fig.6 g;
Felt socks with silken cover and golden decoration	M15:10	Plain weave; hand sewing;	-	21*26*47	Light yellow, brown;	Wool (“*Zhan*”), silk (“*Juan*”), gold;	Golden foil; silk patches;	-	Fig.2 k, l;

Silken fish	M15:11	Plain weave; lap-stitch;	Rectangle;	16.1	Red outside, brown inside;	Silk (“*Bo*”);	-	Tied on the waist of Yingpan Man, left side;	Fig.2 r; Fig.6 j;
Silken gown	M15:12	Plain weave; hand sewing;	Round neck;	110*85	Yellow, green, brown;	Silk (“*Juan*” & “*Jing*”), gold;	Golden foil; silk patches;	Beneath Yingpan Man`s caftan;	Fig.2 k, n;
Perfume sachet	M15:13	Plain weave; hand sewing;		7*7*14	Light yellow, purple, green, red, yellow, brown;	Silk (“*Qi*”), gold;	Golden pendant; silk patches;	Tied on the waist of Yingpan Man, left side;	Fig.2 q; Fig.6 l;
Broken brocade	M15:14	Plain weave;	Rectangle;	20.6~21.3*16.1	Yellow-brown, light brown, navy blue;	Silk (“*Jing*”);	Curved grasses and vines as the main pattern, filled with the images of animals, birds, flowers and Chinese characters of "Shou" and "You";	Placed at the right side of Yingpan Man`s head;	Fig.2 e, k; Fig.6 i; Fig. 7
Felt carpet	M15:15	Hand rolling;	Rectangle;	190*67*0.2	White, yellow;	Fine sheep wool;	-	Beneath Yingpan Man` body;	Fig.2 k;

Table S2. Radiocarbon dating results on the bone from Yingpan Man.

Lab No.	Sample No.	Material	Element	Pretreatment	Conventional Age	Calendar Age*	Median Age
Beta-416250	YPM15-B	Bone collagen	Patella	Collagen extracted with alkali	1730 +/- 30 BP	Cal AD 245-385	305 Cal AD

Note: *Radiocarbon dating results are recalibrated to calendar ages using CALIB REV 7.1.0 with the IntCal13 curve.

Table S3. Stable carbon and nitrogen isotope data of plant samples from Yingpan Cemetery.

Lab No.	Sample No.	Context	Scientific Name	Common Name	Section	Function	No.†	$\delta^{13}C_{VPDB}$ (‰)	SD*	$\delta^{15}N_{AIR}$ (‰)	SD*	%C	SD*	%N	SD*	Atomic C/N
11737	YP-P9	99BYYM66	*Triticum aestivum /Hordeum vulgar*	Wheat/barley	Stalk	Cereal food	4	-26.0	0.3	23.3	0.9	43.4	1.0	0.7	0.1	69.3
11777	YP-P12	99BYYM47	*Triticum aestivum /Hordeum vulgar*	Wheat/barley	Stalk	Cereal food	1	-26.4	-	22.2	-	39.1	-	1.2	-	38.9
11730	YP-P1	95BYYM20	*Sophora alopecuroides var. alopecuroide*	-	Grain	Pillow filler	4	-24.7	0.3	9.0	0.4	45.1	1.3	4.6	0.2	11.5
11731	YP-P2	95BYYM20	*Sophora alopecuroides var. alopecuroide*	-	Stalk	Pillow filler	4	-23.8	0.1	14.1	0.4	44.1	1.0	2.7	0.1	19.1
11732	YP-P3	95BYYM20	*Sophora alopecuroides var. alopecuroide*	-	Stalk	Pillow filler	4	-25.8	0.2	8.0	0.3	42.6	1.2	3.0	0.2	16.7
11735	YP-P7	95BYYM31	*Sophora alopecuroides var. alopecuroide*	-	Grain	Pillow filler	4	-25.5	0.4	10.7	0.4	38.5	0.5	2.9	0.3	15.2
11733	YP-P11	99BYY	*Vitis vinifera*	Grape	Pulp	Fruit/wine making	2	-27.6	-	17.6	-	40.3	-	5.8	-	8.1
11738	YP-P10	99BYY	*Leymus secalinus*	-	Leave	Animal fodder	4	-11.6	0.4	16.9	1.2	35.8	0.8	0.8	0.1	54.0
11733	YP-P5	95BYYM26	*Panicum miliaceum*	Broomcorn millet	Spike	Cereal food/animal fodder	4	-10.7	0.5	9.3	1.4	39.4	0.8	0.8	0.2	54.2
11734	YP-P6	95BYYM26	*Panicum miliaceum*	Broomcorn millet	Stalk	Cereal food/animal fodder	4	-11.5	2.1	9.4	1.9	42.5	1.2	1.0	0.1	49.6
11736	YP-P8	95BYYM26	*Panicum miliaceum*	Broomcorn millet	Stalk	Cereal food/animal fodder	4	-10.7	0.6	9.6	0.7	39.4	0.8	0.8	0.1	57.0
11739	YP-a1	95BYYM26	*Panicum miliaceum*	Broomcorn millet	Caryopsis	Cereal food	2	-9.5	-	10.9	-	38.0	-	2.0	-	22.0

11740	YP-a2	95BYYM26	*Panicum miliaceum*	Broomcorn millet	Palea	Cereal food	2	-10.8	-	11.8	-	34.6	-	0.8	-	48.3
11741	YP-a3	95BYYM26	*Panicum miliaceum*	Broomcorn millet	Lemma	Cereal food	1	-10.7	-	12.0	-	37.4	-	0.8	-	58.1

Note: “-” represents unavailable sample data;

No.† = Number of measurement replication;

SD* is calculated only when sample measurement is over 2 times (≥3).

Table S4. Detailed stable carbon and nitrogen isotope results of plant samples from Yingpan cemetery.

	Lab No.	Sample No.	$\delta^{13}C_{VPDB}$ (‰)	$\delta^{15}N_{AIR}$ (‰)	%C	%N	Atomic C/N
RUN 1	YP-P9	YP-P9	-26.3	23.8	44.3	0.8	62.6
	YP-P1	YP-P1	-24.4	9.0	46.1	4.6	11.7
	YP-P2	YP-P2	-23.9	13.9	44.8	2.8	18.9
	YP-P3	YP-P3	-25.8	7.8	43.8	3.2	16.1
	YP-P7	YP-P7	-26.0	11.0	38.4	3.0	14.7
	YP-P11	YP-P11	-27.6	17.3	40.8	5.9	8.1
	YP-P10	YP-P10	-11.9	15.9	35.6	0.9	48.2
	YP-P04	YP-P04	-11.2	3.0	41.0	1.3	36.8
	YP-P5	YP-P5	-11.1	10.4	40.1	1.0	48.6
	YP-P6	YP-P6	-10.8	10.5	43.3	1.1	47.4
	YP-P8	YP-P8	-11.0	10.0	40.5	0.8	62.7
RUN 2	YP-P9b	YP-P9	-26.1	24.1	44.2	0.9	59.9
	YP-P1b	YP-P1	-24.9	8.8	46.2	4.8	11.2
	YP-P2b	YP-P2	-23.7	14.4	44.8	2.6	20.2
	YP-P3b	YP-P3	-25.8	8.2	43.4	3.0	16.9
	YP-P7b	YP-P7	-25.8	11.1	38.8	2.9	15.9
	YP-P11b	YP-P11	-27.7	17.9	39.7	5.8	8.1
	YP-P10b	YP-P10	-11.8	18.0	36.8	0.8	51.9
	YP-P04b	YP-P5	-11.0	10.4	39.1	1.0	45.9
	YP-P5b	YP-P6	-10.6	11.1	43.6	1.1	48.2
	YP-P6b	YP-P8	-11.3	10.3	39.4	0.9	48.8
RUN 3	11737a	YP-P9	-26.2	22.1	42.5	0.6	80.0
	11730a	YP-P1	-25.0	8.6	44.8	4.5	11.5
	11731a	YP-P2	-23.8	14.5	43.9	2.7	18.8
	11732a	YP-P3	-26.1	7.6	41.6	2.8	17.1
	11735a	YP-P7	-25.1	10.2	39.0	3.3	13.7
	11738a	YP-P10	-11.6	17.8	35.6	0.6	69.3
	11733a	YP-P5	-10.6	7.5	40.0	0.5	89.7
	11734a	YP-P6	-14.6	6.9	42.2	0.9	57.2
	11736a	YP-P8	-10.4	9.2	38.8	0.7	61.1
	11739a	YP-a1	-9.8	10.9	37.3	2.0	21.6

	11740a	YP-a2	-11.3	14.4	36.3	0.8	52.2
	11741a	YP-a3	-10.7	12.0	37.4	0.8	58.2
	11777a	YP-P12	-26.4	22.2	39.1	1.2	38.9
**RUN 4**	11737b	YP-P9	-25.6	23.1	42.5	0.6	77.0
	11730b	YP-P1	-24.4	9.5	43.4	4.3	11.7
	11731b	YP-P2	-23.6	13.6	42.7	2.7	18.8
	11732b	YP-P3	-25.5	8.2	41.5	2.9	16.8
	11735b	YP-P7	-25.3	10.6	37.8	2.6	16.8
	11738b	YP-P10	-11.0	15.7	35.0	0.8	53.5
	11733b	YP-P5	-9.9	8.6	38.3	0.9	50.6
	11734b	YP-P6	-9.9	9.2	40.9	1.0	48.8
	11736b	YP-P8	-9.9	8.8	39.1	0.8	60.9
	11739b	YP-a1	-9.2	10.8	38.7	2.0	22.3
	11740b	YP-a2	-10.2	9.2	32.9	0.9	44.8

Table S5. Stable carbon, nitrogen and sulphur isotope results of faunal samples from Yingpan cemetery.

Lab No.	Sample No.	Context	Species	Element	Description	$\delta^{13}C_{VPDB}$ (‰)	$\delta^{15}N_{AIR}$ (‰)	$\delta^{34}S_{VCDT}$ (‰)	%C	%N	%S	Atomic C/N
AIL002	YP-An1	99BYYM7:2	Goat	Skull	-	-18.4	12.6	-	42.8	15.1	-	3.3
AIL005	YP-An2	99BYYM8:2	Sheep	Rib	-	-18.3	14.9	-	42.0	15.3	-	3.2
AIL003	YP-An3	99BYYM8:2	Sheep	Hair	2-cm long	-17.2	15.4	9.0	47.8	15.0	2.7	3.7
AIL004	YP-An4	99BYYM8:2	Sheep	Meat	Cooked	-17.8	18.7	-	50.7	12.6	-	4.7

Note: “-” represents unavailable sample data.

Table S6. Stable carbon and nitrogen isotope results of serial dentine from the first molar (M1) of Yingpan Man (95BYYM15).

Lab No.	Sample No.	Distance from Crown	Age (yr)*	$\delta^{13}C_{VPDB}$ (‰)	$\delta^{15}N_{AIR}$ (‰)	%C	%N	Atomic C:N
AIL1398	15M1-1	1 mm	0.3	-16.9	19.7	40.7	14.7	3.2
AIL1399	15M1-2	2 mm	0.8	-16.9	19.2	41.9	15.0	3.3
AIL1400	15M1-3	3 mm	1.3	-17.0	18.5	35.4	12.7	3.3
AIL1401	15M1-4	4 mm	1.8	-17.0	17.8	29.6	10.7	3.2
AIL1402	15M1-5	5 mm	2.2	-16.6	17.2	30.1	10.9	3.2
AIL1403	15M1-6	6 mm	2.7	-15.9	17.2	32.7	11.8	3.2
AIL1404	15M1-7	7 mm	3.2	-15.7	17.2	33.5	12.1	3.2
AIL1405	15M1-8	8 mm	3.7	-15.5	17.3	39.4	14.1	3.3
AIL1406	15M1-9	9 mm	4.2	-15.3	17.5	24.4	8.8	3.3
AIL1407	15M1-10	10 mm	4.7	-15.7	17.2	29.6	10.8	3.2
AIL1408	15M1-11	11 mm	5.2	-15.8	17.6	25.2	9.2	3.2
AIL1409	15M1-12	12 mm	5.6	-16.1	17.9	30.0	10.8	3.2
AIL1410	15M1-13	13 mm	6.1	-16.5	17.7	23.7	8.7	3.2
AIL1411	15M1-14	14 mm	6.6	-16.7	17.8	23.3	8.5	3.2
AIL1412	15M1-15	15 mm	7.1	-16.6	17.7	25.5	9.0	3.3
AIL1413	15M1-16	16 mm	7.6	-16.0	17.2	24.5	8.9	3.2
AIL1414	15M1-17	17 mm	8.1	-15.8	17.3	36.1	13.1	3.2
AIL1415	15M1-18	18 mm	8.5	-15.8	17.7	41.8	15.2	3.2
AIL1416	15M1-19	19 mm	9.0	-15.4	17.4	37.3	13.6	3.2
AIL1417	15M1-20	20 mm	9.5	-15.7	17.4	39.8	14.6	3.2
AIL1418	15M1-21	21 mm	10.0	-15.4	17.0	39.3	14.4	3.2

Note: Age* = Approximate Age of Yingpan Man.

Table S7. Averaged stable carbon and nitrogen isotope results of bone collagen samples from Yingpan Man (95BYYM15).

Sample No.	Element	Material	Age*	No.†	$\delta^{13}C_{VPDB}$ (‰)	$\delta^{15}N_{AIR}$ (‰)	%C	%N	Atomic C:N
M15-B	Patella	Bone collagen	~ 5 yrs. before death	2	-15.9	16.5	42.9	16.1	3.1
12030	Mandible	Bone collagen	Childhood	2	-13.7	15.2	38.8	13.2	3.4
12031	Canine	Dentinal collagen	ca. 0.4 - 12 years old	2	-16.4	17.7	31.5	10.4	3.5

Note: Age* = Approximate Age of Yingpan Man;

No.† = Number of measurement replication.

Table S8. Detailed stable carbon and nitrogen isotope results of bone and bulk dentine collagen samples from Yingpan Man (95BYYM15).

Lab No.	Sample No.	Material	Element	$\delta^{13}C_{VPDB}$ (‰)	$\delta^{15}N_{AIR}$ (‰)	C%	N%	Atomic C:N
YPMan-col1	BYY95M15-B	Bone collagen	Patella	-15.8	16.5	42.9	16.1	3.1
YPMan-col2	BYY95M15-B	Bone collagen	Patella	-16.0	16.5	40.0	14.3	3.3
12030 a	BYY95M15-Ma	Bone collagen	Mandible	-13.6	15.3	37.7	12.7	3.5
12030 b	BYY95M15-Ma	Bone collagen	Mandible	-13.5	15.1	55.9	20.0	3.3
12031 a	BYY95M15-C	Dentinal collagen	Canine	-16.4	18.1	34.8	11.2	3.6
12031 b	BYY95M15-C	Dentinal collagen	Canine	-15.7	16.6	43.4	16.0	3.2

Table S9. Averaged stable carbon and nitrogen isotope results of muscle samples from Yingpan Man (95BYYM15).

Sample No.	Material	Estimated Time Before Death	No†	$\delta^{13}C_{VPDB}$ (‰)	SD	$\delta^{15}N_{AIR}$ (‰)	SD	%C	SD	%N	SD	Atomic C:N
M15-Mu1	Muscle	ca. 2 - 3 mon before death	4	-16.7	0.4	19.1	0.4	45.6	0.5	15.2	0.5	3.5
M15-Mu2	Muscle	ca. 2 - 3 mon before death	4	-17.0	0.4	19.9	0.5	42.5	0.5	13.3	0.5	3.7

Note: No.†= Number of measurement replication.

Table S10. Detailed stable carbon and nitrogen isotope results of muscle samples from Yingpan Man (95BYYM15).

Lab No.	Sample No.	$\delta^{13}C_{VPDB}$ (‰)	$\delta^{15}N_{AIR}$ (‰)	%C	%N	Atomic C/N	Comments
M15-Mus1	M15-Mu1	-17.3	20.2	42.3	12.7	3.9	Muscle
M15-Mus2	M15-Mu1	-16.7	20.0	43.1	13.4	3.7	Muscle
M15-Mus3	M15-Mu1	-16.6	19.1	42.3	13.9	3.6	Muscle
M15-Mus4	M15-Mu1	-17.3	20.1	42.0	13.2	3.7	Muscle
M15-Mus5	M15-Mu2	-16.6	19.3	45.4	15.4	3.4	Muscle exterior layer
M15-Mus6	M15-Mu3	-16.5	19.0	45.4	15.5	3.4	Muscle 2nd layer
M15-Mus7	M15-Mu4	-16.5	18.6	45.3	15.3	3.5	Muscle 3rd layer
M15-Mus8	M15-Mu5	-17.4	19.5	46.3	14.4	3.7	Muscle interior layer

Table S11. Averaged stable carbon, nitrogen and sulphur isotope results on hair keratin samples from Yingpan Man (95BYYM15).

Sample No.	Estimated Time Before Death	No.†	$\delta^{13}C_{VPDB}$ (‰)	SD	$\delta^{15}N_{AIR}$ (‰)	SD	$\delta^{34}S_{VCDT}$ (‰)	%C	SD	%N	SD	%S	Atomic C:N	SD	Atomic C/S	Atomic N/S
M15-H1	~ 1 mon before death	1	-16.4	-	15.7	-	10.5	42.9	-	13.6	-	2.7	3.7	-	42.4	11.5
M15-H2	~ 2 mon before death	1	-16.4	-	16.3	-	11.1	41.7	-	13.2	-	3.6	3.7	-	30.9	8.4
M15-H3	~ 3 mon before death	2	-16.4	-	16.3	-	11.2	41.9	-	13.1	-	3.5	3.7	-	31.7	8.5
M15-H4	~ 4 mon before death	3	-16.5	0.3	16.1	0.2	11.3	42.3	1.1	13.5	0.7	3.7	3.7	0.1	30.0	8.1
M15-H5	~ 5 mon before death	3	-16.7	0.5	15.7	0.2	11.4	42.7	2.6	13.7	0.6	3.7	3.6	0.1	30.8	8.3
M15-H6	~ 6 mon before death	3	-16.5	0.4	15.5	0.3	11.3	41.7	1.7	13.4	0.8	3.5	3.6	0.1	30.4	8.2
M15-H7	~ 7 mon before death	3	-15.4	0.2	15.2	0.2	11.4	42.9	1.5	13.9	0.8	3.6	3.6	0.1	31.3	8.6
M15-H8	~ 8 mon before death	3	-14.5	0.1	15.4	0.2	11.5	43.0	2.1	14.0	1.0	3.5	3.6	0.1	31.8	8.7
M15-H9	~ 9 mon before death	3	-14.0	0.1	15.2	0.1	11.4	45.2	3.9	14.8	1.2	3.6	3.6	0.1	36.7	10.2
M15-H10	~ 10 mon before death	3	-14.5	0.3	15.3	0.1	11.3	42.7	1.2	14.0	0.7	3.4	3.6	0.1	33.2	9.1
M15-H11	~ 11 mon before death	3	-15.3	0.3	14.9	0.3	11.0	42.7	1.6	13.9	0.9	3.7	3.6	0.1	30.6	8.4
M15-H12	~ 12 mon before death	3	-16.2	0.4	15.5	0.2	10.9	42.6	1.0	13.9	0.6	3.6	3.6	0.1	31.4	8.6
M15-H13	~ 13 mon before death	3	-16.9	0.2	15.6	0.1	10.6	43.0	1.2	14.1	0.8	3.7	3.6	0.1	30.6	8.4
M15-H14	~ 14 mon before death	3	-17.3	0.2	15.7	0.1	10.6	42.7	1.3	14.0	0.7	3.9	3.6	0.1	28.9	8.0
M15-H15	~ 15 mon before death	3	-16.7	0.3	15.5	0.1	10.7	42.8	0.7	14.0	0.6	3.9	3.6	0.1	28.9	8.0
M15-H16	~ 16 mon before death	3	-16.3	0.2	15.6	0.2	10.4	42.6	1.1	13.9	0.6	3.7	3.6	0.1	30.1	8.4
M15-H17	~ 17 mon before death	3	-15.7	0.2	15.4	0.4	10.8	42.8	0.7	14.0	0.6	3.6	3.6	0.1	31.3	8.6
M15-H18	~ 18 mon before death	3	-15.5	0.1	15.3	0.1	10.5	42.9	0.8	14.0	0.4	3.8	3.6	0.0	29.7	8.3
M15-H19	~ 19 mon before death	3	-15.3	0.2	15.4	0.3	10.6	42.9	1.1	14.0	0.7	3.6	3.6	0.1	31.6	8.7
M15-H20	~ 20 mon before death	3	-15.3	0.2	15.3	0.1	10.7	41.7	0.6	13.6	0.3	3.6	3.6	0.1	31.4	8.7

M15-H21	~ 21 mon before death	3	-15.5	0.1	15.4	0.4	10.9	43.2	1.9	14.1	0.9	3.7	3.6	0.1	30.6	8.5
M15-H22	~ 22 mon before death	3	-15.2	0.2	15.5	0.2	11.2	42.8	1.2	14.0	0.7	3.7	3.6	0.1	30.3	8.3
M15-H23	~ 23 mon before death	3	-15.2	0.5	15.3	0.3	11.6	43.0	1.8	14.1	0.9	3.6	3.6	0.1	31.2	8.6
M15-H24	~ 24 mon before death	3	-15.2	0.2	15.6	0.3	11.1	42.6	0.9	14.0	0.6	4.1	3.6	0.1	27.6	7.6
M15-H25	~ 25 mon before death	3	-15.2	0.2	15.4	0.1	11.5	43.0	1.5	14.1	0.8	4.1	3.6	0.1	27.5	7.6
M15-H26	~ 26 mon before death	3	-15.8	0.6	15.2	0.2	11.4	43.2	1.8	14.1	0.8	4.1	3.6	0.1	27.8	7.6
M15-H27	~ 27 mon before death	3	-15.6	0.6	15.6	0.3	11.4	43.3	1.7	14.3	0.8	4.1	3.5	0.1	28.1	7.8
M15-H28	~ 28 mon before death	3	-16.3	0.4	15.4	0.3	11.6	43.3	1.9	14.2	1.0	4.1	3.6	0.1	27.8	7.6
M15-H29	~ 29 mon before death	3	-16.3	0.2	15.5	0.2	11.5	41.9	0.9	13.8	0.2	4.0	3.5	0.1	28.5	7.9
M15-H30	~ 30 mon before death	3	-16.6	0.3	15.4	0.1	11.5	42.9	1.2	14.0	0.8	3.9	3.6	0.1	29.3	8.0
M15-H31	~ 31 mon before death	3	-16.7	0.6	15.4	0.2	11.3	43.2	0.7	14.1	0.6	4.0	3.6	0.1	28.9	7.9
M15-H32	~ 32 mon before death	3	-16.5	0.3	15.5	0.2	11.3	43.3	1.1	14.1	0.8	3.9	3.6	0.1	29.7	8.1
M15-H33	~ 33 mon before death	3	-16.4	0.2	15.2	0.1	11.3	42.7	1.3	13.9	0.8	3.9	3.6	0.1	29.1	7.9
M15-H34	~ 34 mon before death	3	-16.2	0.3	15.4	0.3	11.7	42.5	0.7	13.8	0.4	3.8	3.6	0.0	29.8	8.2
M15-H35	~ 35 mon before death	3	-16.1	0.2	15.4	0.3	11.7	42.8	1.8	14.0	1.1	3.9	3.6	0.1	28.9	8.0
M15-H36	~ 36 mon before death	3	-15.4	0.6	15.2	0.1	11.5	43.1	1.6	14.1	1.0	3.9	3.6	0.1	29.4	8.1
M15-H37	~ 37 mon before death	3	-15.6	0.4	15.3	0.2	11.8	42.5	1.5	13.9	0.9	3.5	3.6	0.1	32.6	8.9
M15-H38	~ 38 mon before death	3	-15.3	0.2	15.2	0.3	11.9	42.8	1.5	14.0	0.8	3.6	3.6	0.1	31.8	8.8
M15-H39	~ 39 mon before death	3	-15.3	0.2	15.2	0.1	11.9	42.9	1.6	13.9	0.9	3.8	3.6	0.1	30.0	8.1
M15-H40	~ 40 mon before death	3	-15.5	0.1	15.2	0.2	11.8	42.7	1.3	13.9	0.8	3.7	3.6	0.1	30.8	8.5
M15-H41	~ 41 mon before death	3	-15.0	0.2	16.3	1.2	-	47.6	8.8	16.0	4.3	-	3.5	0.3	-	-
M15-H42	~ 42 mon before death	2	-15.3	-	15.5	-	-	42.3	-	13.4	-	-	3.7	-	-	-

M15-H43	~ 43 mon before death	2	-15.0	-	15.0	-		42.4	-	13.5	-	-	3.7	-	-	-
M15-H44	~ 44 mon before death	1	-16.3	-	15.6	-	-	41.7	-	13.3	-	-	3.7	-	-	-
M15-H45	~ 45 mon before death	1	-15.4	-	15.4	-	-	41.9	-	13.5	-	-	3.6	-	-	-
M15-H46	~ 46 mon before death	1	-15.8	-	15.3	-	-	41.9	-	13.4	-	-	3.6	-	-	-

Note: "-" represents unavailable sample data;

No.† = Number of sample replication;

*Samples in grey are excluded for possible contamination.

Table S12. Detailed stable carbon, nitrogen and sulphur isotope results on 3 bundles of hair keratin samples from Yingpan Man (95BYYM15).

		Lab No.	Sample No.	$\delta^{13}C_{VPDB}$ (‰)	$\delta^{15}N_{AIR}$ (‰)	$\delta^{34}S_{VCDT}$ (‰)	C%	N%	S%	Atomic C/N	Atomic C/S	Atomic N/S	Comments
Bundle 1	M15-H3*	AIL001-B1	M15-B1	-15.5	20.2	-	46.0	16.6	-	3.2	-	-	Excluded for possible contamination
	M15-H4	AIL001-B2	M15-B2	-16.2	16.0	-	43.6	14.3	-	3.5	-	-	-
	M15-H5	AIL001-B3	M15-B3	-16.2	15.9	-	40.1	13.2	-	3.5	-	-	-
	M15-H6	AIL001-B4	M15-B4	-16.5	15.8	-	43.3	14.2	-	3.6	-	-	-
	M15-H7	AIL001-B5	M15-B5	-15.2	15.1	-	44.6	14.8	-	3.5	-	-	-
	M15-H8	AIL001-B6	M15-B6	-14.6	15.5	-	45.4	15.2	-	3.5	-	-	-
	M15-H9	AIL001-B7	M15-B7	-14.1	15.1	-	43.9	14.7	-	3.5	-	-	-
	M15-H10	AIL001-B8	M15-B8	-14.9	15.4	-	44.0	14.8	-	3.5	-	-	-
	M15-H11	AIL001-B9	M15-B9	-14.9	14.9	-	44.4	14.9	-	3.5	-	-	-
	M15-H12	AIL001-B10	M15-B10	-16.6	15.5	-	43.6	14.6	-	3.5	-	-	-
	M15-H13	AIL001-B11	M15-B11	-16.7	15.7	-	44.4	15.0	-	3.5	-	-	-
	M15-H14	AIL001-B12	M15-B12	-17.2	15.6	-	44.2	14.8	-	3.5	-	-	-
	M15-H15	AIL001-B13	M15-B13	-16.6	15.6	-	43.6	14.7	-	3.5	-	-	-
	M15-H16	AIL001-B14	M15-B14	-16.4	15.7	-	43.8	14.6	-	3.5	-	-	-
	M15-H17	AIL001-B15	M15-B15	-15.9	15.8	-	43.6	14.6	-	3.5	-	-	-
	M15-H18	AIL001-B16	M15-B16	-15.6	15.4	-	43.8	14.5	-	3.5	-	-	-
	M15-H19	AIL001-B17	M15-B17	-15.4	15.7	-	44.2	14.8	-	3.5	-	-	-
	M15-H20	AIL001-B18	M15-B18	-15.4	15.3	-	41.6	13.9	-	3.5	-	-	-

	M15-H21	AIL001-B19	M15-B19	-15.4	15.8	-	45.3	15.1	-	3.5	-	-	-
	M15-H22	AIL001-B20	M15-B20	-15.4	15.7	-	44.2	14.8	-	3.5	-	-	-
	M15-H23	AIL001-B21	M15-B21	-15.1	15.5	-	45.0	15.2	-	3.5	-	-	-
	M15-H24	AIL001-B22	M15-B22	-15.3	15.9	-	43.6	14.7	-	3.5	-	-	-
	M15-H25	AIL001-B23	M15-B23	-15.1	15.5	-	44.7	15.0	-	3.5	-	-	-
	M15-H26	AIL001-B24	M15-B24	-15.4	15.4	-	45.2	15.0	-	3.5	-	-	-
	M15-H27	AIL001-B25	M15-B25	-15.4	15.9	-	45.0	15.2	-	3.4	-	-	-
	M15-H28	AIL001-B26	M15-B26	-16.6	15.5	-	45.5	15.3	-	3.5	-	-	-
	M15-H29	AIL001-B27	M15-B27	-16.5	15.5	-	41.0	13.9	-	3.4	-	-	-
	M15-H30	AIL001-B28	M15-B28	-16.8	15.4	-	44.2	14.9	-	3.5	-	-	-
	M15-H31	AIL001-B29	M15-B29	-17.3	15.4	-	43.8	14.8	-	3.5	-	-	-
	M15-H32	AIL001-B30	M15-B30	-16.5	15.6	-	44.3	15.0	-	3.4	-	-	-
	M15-H33	AIL001-B31	M15-B31	-16.2	15.1	-	44.0	14.8	-	3.5	-	-	-
	M15-H34	AIL001-B32	M15-B32	-15.9	15.1	-	43.2	14.2	-	3.5	-	-	-
	M15-H35	AIL001-B33	M15-B33	-16.1	15.4	-	44.9	15.2	-	3.4	-	-	-
	M15-H36	AIL001-B34	M15-B34	-14.7	15.1	-	44.8	15.2	-	3.4	-	-	-
	M15-H37	AIL001-B35	M15-B35	-16.0	15.1	-	43.8	14.9	-	3.4	-	-	-
	M15-H38	AIL001-B36	M15-B36	-15.2	14.8	-	44.2	14.9	-	3.5	-	-	-
	M15-H39	AIL001-B37	M15-B37	-15.5	15.1	-	44.5	15.0	-	3.5	-	-	-
	M15-H40	AIL001-B38	M15-B38	-15.4	15.0	-	43.9	14.8	-	3.5	-	-	-
	M15-H41*	AIL001-B39	M15-B39	-15.0	17.7	-	57.7	20.9	-	3.2	-	-	Excluded for possible contamination
	M15-H1*	12194	M15-D1	-16.4	15.7	10.5	42.9	13.6	2.7	3.7	42.4	11.5	

	M15-H2	12195	M15-D2	-16.4	16.3	11.1	41.7	13.2	3.6	3.7	30.9	8.4	-
	M15-H3	12196	M15-D3	-16.4	16.3	11.2	41.6	13.0	3.5	3.7	31.7	8.5	-
	M15-H4	12197	M15-D4	-16.6	16.3	11.3	41.6	13.1	3.7	3.7	30.0	8.1	-
	M15-H5	12198	M15-D5	-17.1	15.6	11.4	42.7	13.4	3.7	3.7	30.8	8.4	-
	M15-H6	12199	M15-D6	-16.1	15.2	11.3	39.9	12.6	3.5	3.7	30.4	8.2	-
	M15-H7	12200	M15-D7	-15.5	15.1	11.4	42.2	13.5	3.6	3.6	31.3	8.5	-
	M15-H8	12201	M15-D8	-14.4	15.1	11.5	41.8	13.3	3.5	3.7	31.8	8.6	-
	M15-H9	12202	M15-D9	-14.0	15.3	11.4	49.6	16.0	3.6	3.6	36.7	10.1	-
	M15-H10	12203	M15-D10	-14.3	15.3	11.3	42.3	13.6	3.4	3.6	33.2	9.1	-
	M15-H11	12204	M15-D11	-15.4	15.1	11.0	42.4	13.6	3.7	3.6	30.6	8.5	-
**Bundle 2**	M15-H12	12205	M15-D12	-15.9	15.4	10.9	42.4	13.6	3.6	3.6	31.4	8.6	-
	M15-H13	12206	M15-D13	-17.1	15.5	10.6	42.4	13.6	3.7	3.6	30.6	8.3	-
	M15-H14	12207	M15-D14	-17.2	15.7	10.6	42.2	13.6	3.9	3.6	28.9	8.0	-
	M15-H15	12208	M15-D15	-16.5	15.5	10.7	42.3	13.6	3.9	3.6	28.9	8.0	-
	M15-H16	12209	M15-D16	-16.3	15.7	10.4	41.7	13.6	3.7	3.6	30.1	8.4	-
	M15-H17	12210	M15-D17	-15.7	15.4	10.8	42.3	13.6	3.6	3.6	31.3	8.6	-
	M15-H18	12211	M15-D18	-15.5	15.2	10.5	42.3	13.8	3.8	3.6	29.7	8.3	-
	M15-H19	12212	M15-D19	-15.1	15.4	10.6	42.6	13.7	3.6	3.6	31.6	8.7	-
	M15-H20	12213	M15-D20	-15.0	15.4	10.7	42.4	13.7	3.6	3.6	31.4	8.7	-
	M15-H21	12214	M15-D21	-15.6	15.1	10.9	42.5	13.7	3.7	3.6	30.6	8.5	-
	M15-H22	12215	M15-D22	-15.2	15.5	11.2	42.1	13.5	3.7	3.6	30.3	8.3	-
	M15-H23	12216	M15-D23	-15.7	15.4	11.6	42.1	13.5	3.6	3.6	31.2	8.6	-

	M15-H24	12217	M15-D24	-15.3	15.4	11.1	42.4	13.7	4.1	3.6	27.6	7.6	-
	M15-H25	12218	M15-D25	-15.1	15.5	11.5	42.3	13.6	4.1	3.6	27.5	7.6	-
	M15-H26	12219	M15-D26	-16.4	15.0	11.4	42.8	13.7	4.1	3.6	27.8	7.7	-
	M15-H27	12220	M15-D27	-16.2	15.4	11.4	43.2	14.0	4.1	3.6	28.1	7.8	-
	M15-H28	12221	M15-D28	-16.3	15.1	11.6	42.7	13.7	4.1	3.6	27.8	7.7	-
	M15-H29	12222	M15-D29	-16.4	15.3	11.5	42.8	13.9	4.0	3.6	28.5	7.9	-
	M15-H30	12223	M15-D30	-16.6	15.3	11.5	42.8	13.7	3.9	3.6	29.3	8.1	-
	M15-H31	12224	M15-D31	-16.2	15.2	11.3	43.4	13.9	4.0	3.6	28.9	8.1	-
	M15-H32	12225	M15-D32	-16.3	15.3	11.3	43.4	13.9	3.9	3.6	29.7	8.1	-
	M15-H33	12226	M15-D33	-16.6	15.2	11.3	42.6	13.5	3.9	3.6	29.1	7.8	-
	M15-H34	12227	M15-D34	-16.5	15.6	11.7	42.4	13.7	3.8	3.7	29.8	8.3	-
	M15-H35	12228	M15-D35	-15.9	15.6	11.7	42.2	13.6	3.9	3.6	28.9	8.0	-
	M15-H36	12229	M15-D36	-15.8	15.3	11.5	43.0	13.8	3.9	3.6	29.4	8.1	-
	M15-H37	12230	M15-D37	-15.2	15.3	11.8	42.8	13.7	3.5	3.7	32.6	9.0	-
	M15-H38	12231	M15-D38	-15.1	15.4	11.9	42.9	13.9	3.6	3.6	31.8	8.9	-
	M15-H39	12232	M15-D39	-15.2	15.2	11.9	42.8	13.5	3.8	3.7	30.0	8.0	-
	M15-H40	12233	M15-D40	-15.5	15.3	11.8	42.8	13.7	3.7	3.6	30.8	8.6	$\delta^{34}S$ value excluded for low peak
	M15-H41	12234	M15-D41	-14.8	15.5	-	42.9	13.7	-	3.7	-	-	-
	M15-H42	12235	M15-D42	-15.2	15.3	-	42.8	13.6	-	3.7	-	-	
	M15-H43	12236	M15-D43	-14.7	14.8	-	43.0	13.6	-	3.7	-	-	-
**Bundle 3**	M15-H3	12237	M15-E01	-16.4	16.3	-	42.1	13.1	-	3.7	-	-	-
	M15-H4	12238	M15-E02	-16.8	15.9	-	41.8	13.2	-	3.7	-	-	-

	M15-H5	12239	M15-E03	-16.8	15.7	-	45.2	14.4	-	3.7	-	-	-
	M15-H6	12240	M15-E04	-16.8	15.4	-	42.0	13.4	-	3.7	-	-	-
	M15-H7	12241	M15-E05	-15.6	15.5	-	41.9	13.5	-	3.6	-	-	-
	M15-H8	12242	M15-E06	-14.4	15.5	-	41.8	13.5	-	3.6	-	-	-
	M15-H9	12243	M15-E07	-14.0	15.2	-	42.1	13.6	-	3.6	-	-	-
	M15-H10	12244	M15-E08	-14.4	15.2	-	41.8	13.5	-	3.6	-	-	-
	M15-H11	12245	M15-E09	-15.5	14.6	-	41.2	13.2	-	3.8	-	-	-
	M15-H12	12246	M15-E10	-16.1	15.7	-	41.7	13.6	-	3.6	-	-	-
	M15-H13	12247	M15-E11	-17.0	15.7	-	42.2	13.6	-	3.6	-	-	-
	M15-H14	12248	M15-E12	-17.5	15.7	-	41.8	13.5	-	3.6	-	-	-
	M15-H15	12249	M15-E13	-17.1	15.4	-	42.6	13.8	-	3.6	-	-	-
	M15-H16	12250	M15-E14	-16.1	15.4	-	42.2	13.6	-	3.6	-	-	-
	M15-H17	12251	M15-E15	-15.5	15.1	-	42.4	13.7	-	3.6	-	-	-
	M15-H18	12252	M15-E16	-15.4	15.3	-	42.6	13.8	-	3.6	-	-	-
	M15-H19	12253	M15-E17	-15.5	15.2	-	42.0	13.6	-	3.6	-	-	-
	M15-H20	12254	M15-E18	-15.4	15.3	-	41.2	13.3	-	3.6	-	-	-
	M15-H21	12255	M15-E19	-15.4	15.2	-	41.7	13.5	-	3.6	-	-	-
	M15-H22	12256	M15-E20	-15.0	15.4	-	42.0	13.6	-	3.6	-	-	-
	M15-H23	12257	M15-E21	-14.8	15.0	-	41.8	13.7	-	3.6	-	-	-
	M15-H24	12258	M15-E22	-14.9	15.5	-	41.8	13.5	-	3.6	-	-	-
	M15-H25	12259	M15-E23	-15.5	15.3	-	42.0	13.6	-	3.6	-	-	-
	M15-H26	12260	M15-E24	-15.5	15.1	-	41.7	13.5	-	3.6	-	-	-

	M15-H27	12261	M15-E25	-15.1	15.6	-	41.7	13.6	-	3.6	-	-	-
	M15-H28	12262	M15-E26	-15.9	15.6	-	41.8	13.5	-	3.6	-	-	-
	M15-H29	12263	M15-E27	-16.1	15.6	-	41.8	13.5	-	3.6	-	-	-
	M15-H30	12264	M15-E28	-16.3	15.5	-	41.8	13.4	-	3.6	-	-	-
	M15-H31	12265	M15-E29	-16.6	15.5	-	42.5	13.6	-	3.6	-	-	-
	M15-H32	12266	M15-E30	-16.8	15.5	-	42.1	13.5	-	3.6	-	-	-
	M15-H33	12267	M15-E31	-16.3	15.3	-	41.4	13.3	-	3.6	-	-	-
	M15-H34	12268	M15-E32	-16.3	15.4	-	41.8	13.4	-	3.6	-	-	-
	M15-H35	12269	M15-E33	-16.2	15.1	-	41.4	13.2	-	3.7	-	-	-
	M15-H36	12270	M15-E34	-15.6	15.3	-	41.6	13.3	-	3.7	-	-	-
	M15-H37	12271	M15-E35	-15.5	15.4	-	40.9	13.1	-	3.6	-	-	-
	M15-H38	12272	M15-E36	-15.5	15.3	-	41.3	13.3	-	3.6	-	-	-
	M15-H39	12273	M15-E37	-15.2	15.3	-	41.4	13.3	-	3.6	-	-	-
	M15-H40	12274	M15-E38	-15.5	15.4	-	41.4	13.3	-	3.6	-	-	-
	M15-H41	12275	M15-E39	-15.2	15.7	-	42.1	13.3	-	3.7	-	-	-
	M15-H42	12276	M15-E40	-15.4	15.7	-	41.8	13.2	-	3.7	-	-	-
	M15-H43	12277	M15-E41	-15.2	15.2	-	41.8	13.4	-	3.6	-	-	-
	M15-H44	12278	M15-E42	-16.3	15.6	-	41.7	13.3	-	3.7	-	-	-
	M15-H45	12279	M15-E43	-15.4	15.4	-	41.9	13.5	-	3.6	-	-	-
	M15-H46	12280	M15-E44	-15.8	15.3	-	41.9	13.4	-	3.7	-	-	-

Note: “-” represents unavailable sample data;

*Samples in grey are excluded for possible contamination.

Table S13. Details of the results of the isotopic mixing model of FRUITS (estimated % of contribution from different food sources).

Scenario/Sample	Source/Food	Isotopic Data						FRUITS				
		N	Element	$\delta^{13}C$ (‰)	Unc.	$\delta^{15}N$ (‰)	Unc.	Mean	SD	2.5pc	median	97.5pc
A ($\delta^{13}C$=-14.0±0.1 δ15N=15.2±0.1)	Millet	3	Grain	-10.3	1	11.6	1	53%	7%	39%	53%	67%
	Wheat/barley	2	Stalk	-24.9	1	20.4	1	16%	15%	0%	12%	51%
	Grape	1	Pulp	-27.6	1	17.6	1	24%	13%	1%	25%	46%
	Sheep/goat	2	Bone	-18.4	1	13.8	1	7%	8%	0%	4%	30%
B ($\delta^{13}C$=-17.3±0.2 δ15N=15.7±0.1)	Millet	3	Grain	-10.3	1	11.6	1	28%	9%	8%	28%	44%
	Wheat/barley	2	Stalk	-24.9	1	20.4	1	26%	21%	1%	21%	71%
	Grape	1	Pulp	-27.6	1	17.6	1	21%	15%	1%	19%	48%
	Sheep/goat	2	Bone	-18.4	1	13.8	1	25%	27%	0%	9%	81%

Notes: The isotopic fractionation between wheat/barley stalk and grain is corrected with an offset of +1.3‰ for $\delta^{13}C$ values[3] and –2.4‰ for $\delta^{15}N$ values[4].

References

1 Zhou, J., Li, W., Nijiati & Hasiyeti. Brief report on the excavation of tomb M15 of Yingpan Cemetery, Weili, Xinjiang. *cultural relics*, 97-102 (1999).

2 Chen, T., Wang, X., Dai, J., Li, W. & Jiang, H. Plant use in the Lop Nor region of southern Xinjiang, China: Archaeobotanical studies of the Yingpan cemetery (~ 25–420 AD). *Quaternary International* **426**, 166-174 (2016).

3 Zhao, F.-J., Baruch, S. & McGrath, S. P. Trends in 13C/12C ratios and C isotope discrimination of wheat since 1845. *Oecologia* **128**, 336-342 (2001).

4 Fraser, R. A. *et al.* Manuring and stable nitrogen isotope ratios in cereals and pulses: towards a new archaeobotanical approach to the inference of land use and dietary practices. *J. Archaeol. Sci.* **38**, 2790-2804, doi:http://dx.doi.org/10.1016/j.jas.2011.06.024 (2011).
